# A Nonadjuvanted Whole-Inactivated Pneumococcal Vaccine Induces Multiserotype Opsonophagocytic Responses Mediated by Noncapsule-Specific Antibodies

**DOI:** 10.1128/mbio.02367-22

**Published:** 2022-09-20

**Authors:** Shannon C. David, Erin B. Brazel, Eve V. Singleton, Vikrant Minhas, Zoe Laan, Catherine Scougall, Austen Y. Chen, Hui Wang, Chloe J. Gates, Kimberley T. McLean, Jeremy S. Brown, Giuseppe Ercoli, Rachel A. Higgins, Paul V. Licciardi, Kim Mulholland, Justin B. Davies, Timothy R. Hirst, James C. Paton, Mohammed Alsharifi

**Affiliations:** a Research Centre for Infectious Diseases (RCID), and Department of Molecular and Biomedical Sciences, The University of Adelaide, SA, Australia; b GPN Vaccines, Yarralumla, ACT, Australia; c Centre for Inflammation and Tissue Repair, UCL Respiratory, University College London, London, United Kingdom; d New Vaccines, Murdoch Children’s Research Institute, Parkville, VIC, Australia; e Department of Paediatrics, University of Melbourne, VIC Australia; f Irradiations Group, Australian Nuclear Science and Technology Organization (ANSTO), Lucas Heights, NSW, Australia; University of Mississippi Medical Center

**Keywords:** *Streptococcus pneumoniae*, adjuvants, *in vivo* protection, inactivated whole-cell vaccine, serotype independent protection, opsonophagocytic response, vaccines

## Abstract

Streptococcus pneumoniae (Spn) remains a major cause of global mortality, with extensive antigenic diversity between capsular serotypes that poses an ongoing challenge for vaccine development. Widespread use of pneumococcal conjugate vaccines (PCVs) targeting Spn capsules has greatly reduced infections by vaccine-included serotypes but has led to increased infections by nonincluded serotypes. To date, high cost of PCVs has also limited their usefulness in low-income regions where disease burdens are highest. To overcome these limitations, serotype-independent vaccines are being actively researched. We have developed a whole-cell gamma-irradiated Spn vaccine (termed Gamma-PN) providing serotype-independent protection. We demonstrate that Gamma-PN immunization of mice or rabbits via the clinically relevant intramuscular route induces protein-specific antibodies able to bind numerous nonvaccine encapsulated serotypes, which mediate opsonophagocytic killing and protection against lethal challenges. Gamma-PN induced comparable or superior opsonophagocytic killing assay (OPKA) responses in rabbits to the licensed Prevnar 13 vaccine (PCV13) for vaccine-included serotypes, and a superior response to nonincluded serotypes, including emergent 22F and 35B. Additionally, despite a lower observed reactogenicity, administration of Gamma-PN without adjuvant resulted in higher OPKA responses and improved protection compared to adjuvanted Gamma-PN. To our knowledge, this has not been demonstrated previously for a whole-inactivated Spn vaccine. Eliminating the requirement for adjuvant comes with numerous benefits for clinical applications of this vaccine and poses interesting questions for the inclusion of adjuvant in similar vaccines in development.

## INTRODUCTION

Streptococcus pneumoniae (Spn) is an encapsulated diplococcus forming part of the commensal microbiome in the human nasopharynx. Carriage is typically asymptomatic, although Spn can translocate to other body sites to cause pneumonia and other invasive pneumococcal diseases (IPD). A recent review concluded Spn remains the most common cause of community-acquired pneumonia and is the most frequently isolated pathogen from patients with recurrent pneumonia (1). Spn is also notorious for causing secondary infection in patients with respiratory viral infections, leading to more severe disease and heightened mortality. While not explicitly linked to Spn, secondary bacterial infection of COVID patients was identified as a strong predictor of death (2).

Spn is classified into antigenically distinct serotypes based on the chemical structure of the capsule, with 100 unique serotypes currently identified (3). Capsular polysaccharides (CPS) are a major Spn virulence factor and are the primary antigen targets for current pneumococcal conjugate vaccines (PCVs). While effective, PCVs cover a limited selection of serotypes (e.g., 13 serotypes covered by PCV13, 20 serotypes by the newly licensed PCV20). This has led to rapid serotype replacement in multiple regions (4–6), with many emerging serotypes also being antibiotic-resistant (7–10). Indeed, the 3rd, 4th, and 5th most prevalent serotypes responsible for IPD in the US are not covered by PCV20, rendering the vaccine of limited utility. Furthermore, Spn serotype switching can occur via genetic transfer of capsule-related genes. Almost all Spn genes necessary for capsule biosynthesis are contained in a genetic cassette (*cps*), and a new Spn strain may arise after capturing genetic material from a nonvaccine Spn strain or a related species like oral streptococci (11). Indeed, analysis of the 100th unique serotype, 10D (Ganaie et al. [3]) provided compelling evidence for interspecies genetic transfer of *cps* from oral streptococci to pneumococci. Since oral streptococci have a large repertoire of *cps* loci, ongoing usage of capsule-specific Spn vaccines could drive emergence of more novel Spn serotypes through interspecies recombination.

These pressing issues paired with the cost and complexity of PCV manufacture have led to the pursuit of alternative vaccine strategies, including protein and whole-cell vaccine (WCV) candidates. WCVs present an appealing solution as they can be relatively inexpensively manufactured and present an almost complete range of Spn antigens in native conformation to the recipient’s immune system. Redundancy of protein expression increases the likelihood that WCV-induced responses are reactive against a multitude of pneumococcal strains regardless of capsular type, isolation site, and genetic background. It has also been shown that naturally acquired protection against IPD largely depends on antibodies against protein antigens rather than capsule in mice (12) and humans (13). The WCV approach has been pursued by several groups using live-attenuated bacteria (12, 14), bacterial lysates (15), or inactivated whole-cell preparations (16–20).

Our prior studies detailed the development (18) and optimization (20) of a WCV comprising unencapsulated Spn bacteria inactivated by gamma irradiation (Gamma-PN). These prior studies demonstrated protection after intranasal immunization with no requirement for a mucosal adjuvant (18, 21). However, given that aluminum-based adjuvants are widely used in commercial vaccines, including PCV13, we sought to investigate the effect of an approved adjuvant on Gamma-PN efficacy. The intramuscular (IM) route was utilized in this study to investigate the effect of aluminum hydroxide adjuvant (Alhydrogel), and to investigate noninferiority of Gamma-PN (as a WCV) compared to the CPS-based PCV13.

## RESULTS

### Nonadjuvanted Gamma-PN shows high protective efficacy against Spn challenge.

**FIG 1:**
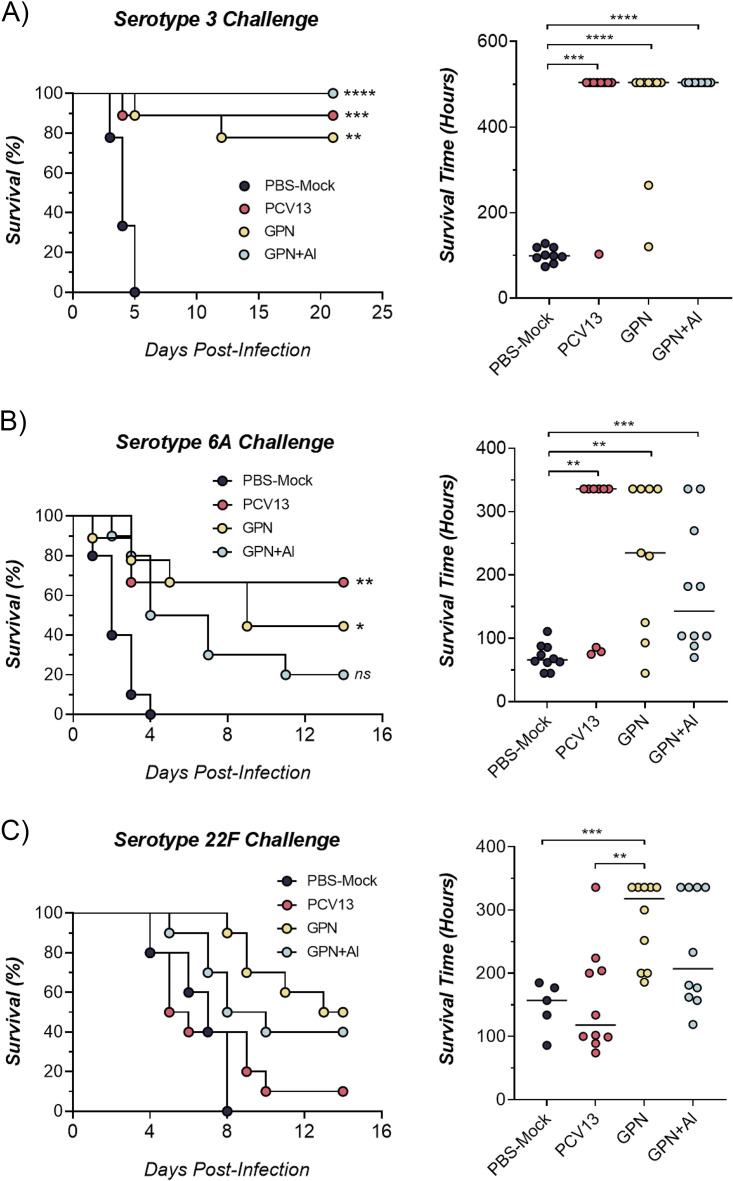
Protective efficacy induced by Gamma-PN and PCV13. Swiss mice were vaccinated IM with Gamma-PN alone or Gamma-PN+Al (25 μg total protein of Gamma-PN per dose [A], and 50 μg per dose for [B and C]). Control mice received the commercially available PCV13 vaccine IM, or PBS+Al IM as a mock vaccine control. All mice received 3 immunizations administered 2 weeks apart. Two weeks after the final immunization, all mice were anaesthetized and challenged IN with either (A) serotype 3 (2 × 10^7^ CFU/mouse), (B) serotype 6A (1 × 10^8^ CFU/mouse), or (C) serotype 22F (1 × 10^8^ CFU/mouse). Mice were monitored multiple times daily for development of clinical symptoms and euthanized once they became moribund. For all experiments, *n* = 8 to 10 mice/group, except for the PBS mock control group (C), where *n* = 5. Data presented as total survival percentages (analyzed by Fisher exact test, compared to PBS mock control group) and as median survival time (analyzed by Mann-Whitney test), ****, *P* < 0.0001; ***, *P* < 0.001; **, *P* < 0.01; *, *P* < 0.05; ns, not significant. Data representative of 2 (A and B) or 1 (C) independent experiments.

The Gamma-PN vaccine is derived from the nonencapsulated Rx1 strain previously modified to delete the autolysin (*lytA*) gene and attenuate the pneumolysin (*ply*) gene (detailed in Babb et al. [18]), and to delete the pneumococcal surface antigen A (*psaA*) gene (detailed in David et al. [20]); this strain was then inactivated by gamma irradiation (see Materials and Methods). Initially, we sought to demonstrate noninferiority of Gamma-PN against PCV13 for protection against lethal Spn challenges. Mice were immunized intramuscularly (IM) with three doses of either PCV13, Gamma-PN alone, or Gamma-PN in combination with the adjuvant Alhydrogel (Gamma-PN+Al), then challenged IN with Spn serotypes 3, 6A, or 22F (all heterologous serotypes to the ancestor of Gamma-PN). Fig 1A demonstrates Gamma-PN alone and Gamma-PN+Al were equivalent to PCV13 for protection against challenge with the highly encapsulated serotype 3, with all vaccines inducing significant protection compared to phosphate-buffered saline (PBS) mock controls. The second PCV13-included serotype, 6A (Fig. 1B) showed similar protection rates between Gamma-PN alone and PCV13, with survival rates of 50% and 70%, respectively (no significant difference by Fisher Exact test or Mann-Whitney U-test). Interestingly, however, IM vaccination with Gamma-PN+Al conferred significant protection against serotype 6A in terms of survival time, but not survival percentage (survival not significantly higher than PBS mock controls). The lower efficacy of Gamma-PN+Al relative to Gamma-PN alone was further apparent when mice were challenged with serotype 22F (Fig. 1C), as only Gamma-PN alone afforded a significant increase in survival time compared to PBS mock controls. Importantly, nonadjuvanted Gamma-PN was significantly more effective than PCV13 for protection against this non-PCV13 serotype.

### Adjuvant increases reactogenicity at the injection site.

**FIG 2:**
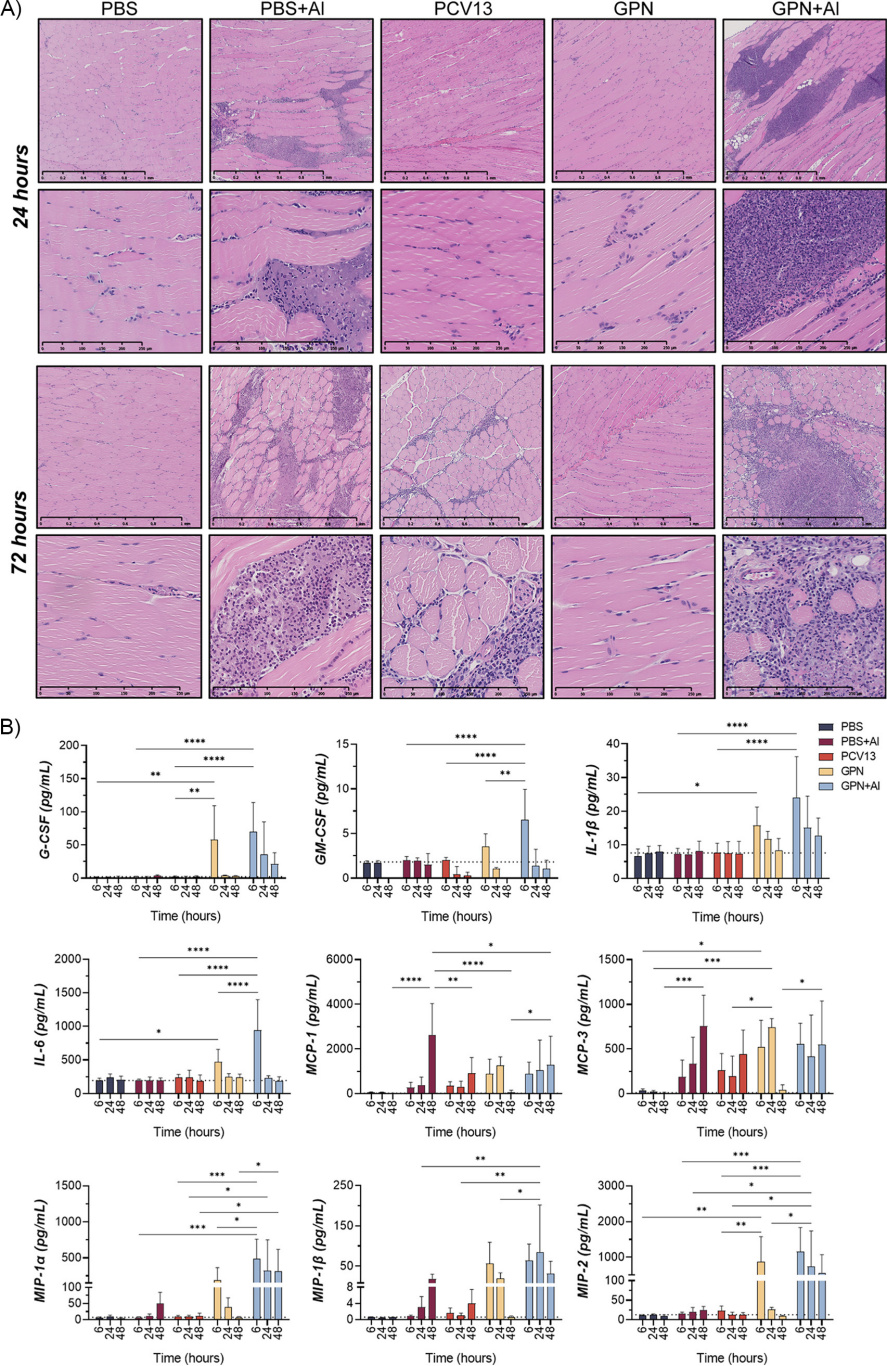
Analysis of inflammatory responses at the immunization site. Swiss mice were vaccinated IM with Gamma-PN alone or Gamma-PN+Al (25 μg of Gamma-PN/dose in both cases), or the commercially available PCV13 vaccine. Control mice received PBS or PBS+Al IM as a mock vaccine. All mice received one dose of vaccine in each quadricep muscle as a singular immunization event. (A) At 24 or 48 h postimmunization, left quadricep muscles were excised and treated for histology. Muscles were fixed in 10% neutral buffered formalin, then embedded in paraffin blocks and sectioned. Sections were H&E stained and imaged with a light microscope. Images are representative of each immunization group (*n* = 5 mice per group) from one experiment. For each time point, scalebars = 1 mm (top) and 250 μm (bottom). (B) At 6, 24, and 48 h, right quadricep muscles were excised and treated for cytokine analysis. Muscles were homogenized in 1 mL PBS, then centrifuged and supernatant was analyzed by cytokine multiplex assay. Data presented as mean cytokine concentration ± SD (*n* = 5 mice per group), analyzed by two-way ANOVA (****, *P* < 0.0001; ***, *P* < 0.001; **, *P* < 0.01; *, *P* < 0.05; any comparison between appropriate groups not shown on the graph was not significant). Dotted line indicates the mean pg/mL for each cytokine in the muscle of nontreated control mice (*n* = 3). Data are representative of two independent cytokine multiplex experiments.

It is well-known that aluminum-containing adjuvants induce inflammation at the injection site, leading to recruitment of antigen-presenting cells. As the addition of adjuvant to Gamma-PN had some effects on protective efficacy, it became important to address the impact of adjuvant on inflammatory responses. To investigate the kinetics of innate immune cell influx, mice were IM immunized once with Gamma-PN, Gamma-PN+Al, or PCV13, and muscle samples were harvested 6 to 72 h postvaccination for histology and cytokine analyses. These time points were chosen based on previous studies reporting local edema and infiltration of neutrophils at 24 to 72 h after IM injection of similarly adjuvanted vaccines (22, 23), with appearance of monocytes and dendritic cell precursors by 72 h (24). Hematoxylin and eosin staining revealed rapid inflammatory responses in muscles injected with Gamma-PN+Al, indicated by infiltration of polymorphonuclear leukocytes by 24 h postimmunization (Fig. 2A). Pockets of densely populated nucleated cells were visible in muscles injected with Gamma-PN+Al and with PBS+Al (control), while nucleated cells in muscles treated with Gamma-PN and PCV13 were far less dense and more evenly dispersed throughout the tissue. Additionally, some necrosis of myocytes was visible in muscles injected with Gamma-PN+Al and PBS+Al (indicated by the thinning/waving of muscle fibers and an increase in intercellular spaces), suggesting muscle damage in these groups.

To further investigate the innate immune landscape at the injection site, muscle homogenates were analyzed by cytokine multiplex assay (Crux Biolabs, Melbourne, Australia). Fig 2B shows vaccination with Gamma-PN+Al induced the most pronounced and prolonged inflammatory state of the five groups. As early as 6 h postvaccination, Gamma-PN+Al induced significantly elevated levels of inflammatory cytokines GM-CSF, IL-6, and MIP-1α compared to Gamma-PN only or control groups. Additional cytokines G-CSF, IL-1β, MCP-1, MCP-3, MIP-1β, and MIP-2 were comparable between the Gamma-PN and Gamma-PN+Al groups at 6 h but remained elevated in the Gamma-PN+Al group only for the full-time course. This is partially attributed to adjuvant, as PBS+Al controls showed increasing levels of inflammatory mediators MCP-1, MCP-3, and MIP-1β with time. The cytokine profile of Gamma-PN alone largely resembled that of PCV13 at 24 and 48 h, except for MCP-3 and MIP-1β, which were higher in Gamma-PN treated mice at 24 h.

### Adjuvant alters serum IgG subclass profiles in mice.

**FIG 3:**
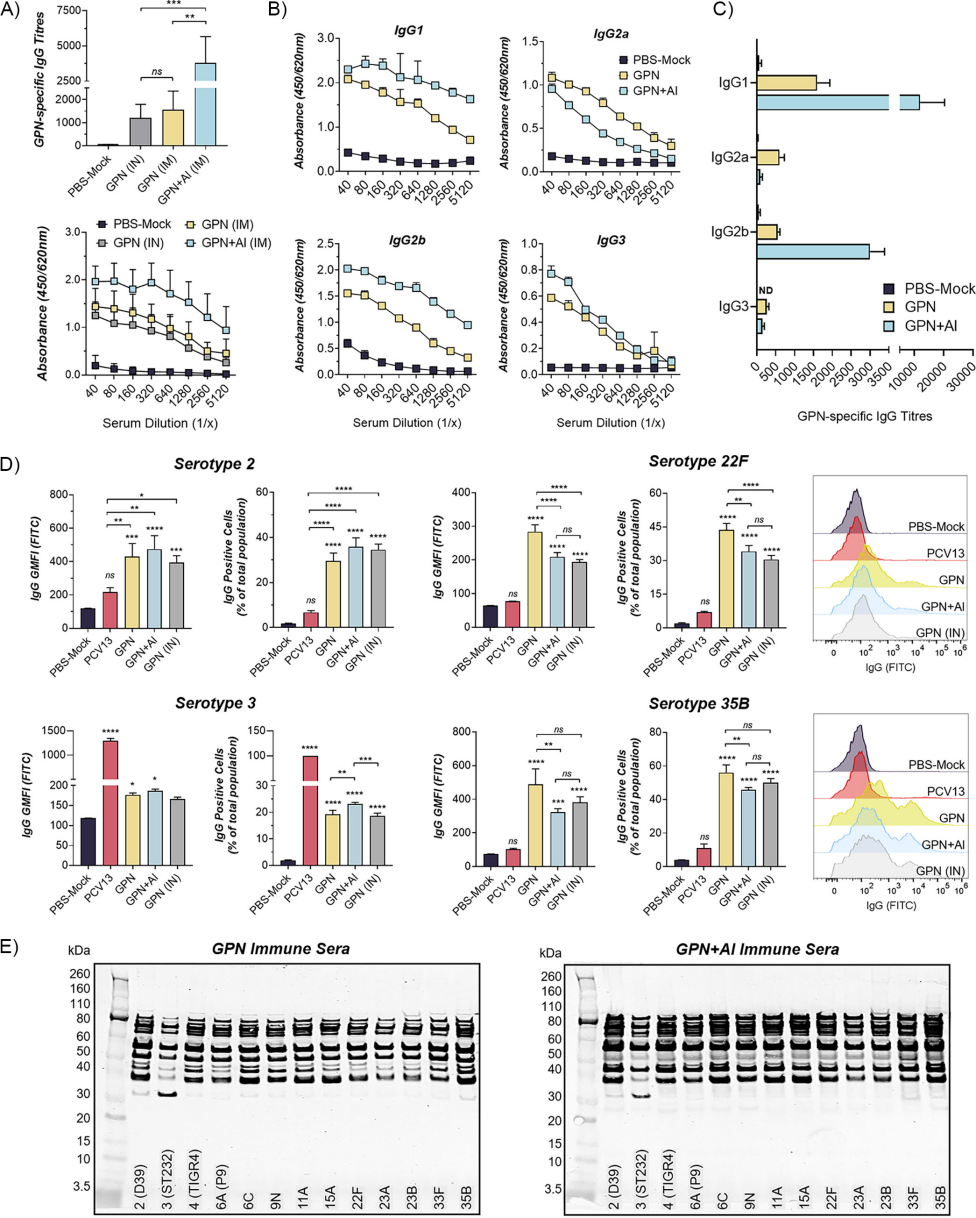
The effect of adjuvant on IgG antibody responses after Gamma-PN immunization of mice. Swiss mice were vaccinated IM with Gamma-PN or Gamma-PN+Alhydrogel (+Al) (25 μg Gamma-PN/dose in both cases). For IN vaccination, animals received 25 μg of Gamma-PN alone (no Al). Control mice received PBS+Al IM as a mock vaccine. All mice received 3 immunizations administered 2 weeks apart, and sera were harvested 1 week after the final immunization. (A) Total Spn-specific IgG in sera of immunized and control mice was determined by ELISA, using whole-cell Gamma-PN as coating antigen. Data presented as mean titers ± SD, and as mean absorbance values ± SD (*n* = 10 mice per group). Titers were calculated using nonlinear regression modeling and analyzed by one-way ANOVA (***, *P* < 0.001; **, *P* < 0.01; ns, not significant). (B) Absorbance values for the subclasses IgG1, IgG2a, IgG2b, and IgG3 for each IM immunization group were determined by ELISA, using Gamma-PN as coating antigen (*n* = 3 mice per group, mean absorbance ± SD is shown). (C) Relative serum titers for each IgG subclass are also presented (mean ± SD). Data generated from two independent experiments. ND, not detected. (D) Intact Spn strains of serotype 2, 3, 22F, and 35B were probed with harvested murine sera (pooled within each vaccine group, *n* = 10 mice per group), then washed and stained with antimurine IgG FITC-conjugated antibody. Pooled immune sera from mice (*n* = 10) immunized IM 3× with PCV13 was included as an additional binding control. All Spn samples were acquired on an Accuri flow cytometer, with a minimum of 10,000 events acquired per sample. Data are presented as FITC GMFI (mean ± SD) of ungated samples, and as percentage of the total bacterial population staining positive for IgG (mean ± SD) relative to unstained controls. Representative histograms for FITC fluorescence are also shown for serotypes 22F and 35B. Data are compiled from at least two independent flow cytometry experiments for all serotypes, with pooled sera tested in technical triplicate in each case. Data were analyzed by one-way ANOVA, with significance relative to PBS mock controls indicated on top of each column, and significance between vaccine groups indicted by horizontal lines (****, *P* < 0.0001; ***, *P* < 0.001; **, *P* < 0.01; *, *P* < 0.05; ns, not significant). (E) Reactivity of pooled murine sera from Gamma-PN or Gamma-PN+Al IM immunized mice was tested by Western Blot against whole-cell lysates of a range of Spn serotypes. Western blots are representative of two independent tests of pooled immune sera. Note that both blots were probed with immune sera at a set dilution of 1/1,000, hence all bands appeared more intense for the Gamma-PN+Al group due to the higher overall IgG titer.

Gamma-PN+Al appeared to induce higher reactogenicity, but lower protection rates compared to Gamma-PN alone and PCV13. This suggested adjuvant was interfering with induction of adaptive responses against Spn, namely, humoral immunity. To investigate the effect of adjuvant on Gamma-PN-specific antibodies, mice were immunized IM three times with Gamma-PN alone or Gamma-PN+Al. Mice were also immunized with Gamma-PN via the intranasal (IN) route as an additional control. Immune sera were harvested from all mice after the 3rd immunization, and total IgG titers and the relative abundance of murine IgG subclasses (IgG1, IgG2a, IgG2b, and IgG3) were determined by ELISA. Total IgG titers were comparable when Gamma-PN was administered via IN and IM routes without adjuvant, indicating the choice of parenteral or mucosal immunization route did not negatively affect antibody responses (Fig. 3A). As anticipated, the addition of adjuvant to IM-administered Gamma-PN (Gamma-PN+Al) resulted in a substantial increase in serum IgG, and a marked difference in IgG subclass profiles (Fig. 3B and C). Spn-specific IgG1 and IgG2b were enhanced by adjuvant, while IgG2a was somewhat reduced. IgG3 did not appear to be affected. Importantly, the addition of adjuvant altered these subclass ratios, with the ratio of IgG1:IgG2a and IgG1:IgG3 being higher in Gamma-PN+Al immunized mice compared to Gamma-PN alone (see Table S3 in Supplemental Material). This suggests an adjuvant-mediated shift toward Th2-type immunity which favors IgG1 over other subclasses.

10.1128/mbio.02367-22.8 TABLE S3 Ratios of serum IgG subclasses for mice immunized IM with Gamma-PN or Gamma-PN+Alhydrogel (+Al). Immune sera were analyzed using subclass specific ELISA’s, and IgG1, IgG2a, IgG2b, and IgG3 titers were calculated using nonlinear regression modelling (EC_50_ method, as described in Materials and Methods). Ratios of each subclass pair were calculated for individual mice (*n* = 3 per group), and data are presented as the mean ratio (± standard deviation) for each vaccine group. Data analyzed by two-way ANOVA with Sidak’s correction for multiple comparisons to assess significant ratio changes between the two vaccine groups (*, *P < *0.05). Download Table S3, DOCX file, 0.01 MB. Copyright © 2022 David et al. 2022 David et al. https://creativecommons.org/licenses/by/4.0/ This content is distributed under the terms of the Creative Commons Attribution 4.0 International license.

### Gamma-PN induces cross-reactive antibodies against Spn proteins in mice.

The ability of Gamma-PN-induced antibodies to bind whole-encapsulated Spn bacteria was then assessed using flow cytometry. Immune sera from mice immunized with PCV13 were also included here to compare functionality of WCV-induced antibodies to those induced by the CPS-based PCV13. Multiple serotypes of live encapsulated Spn were incubated with immune sera, then incubated with a fluorescein isothiocyanate (FITC)-conjugated antimurine IgG antibody and analyzed for IgG binding by flow cytometry.

Fig 3D shows comparable binding by sera from animals vaccinated with Gamma-PN alone by IN and IM routes, again indicating Gamma-PN was just as immunogenic when administered IM. Furthermore, Gamma-PN-induced antibodies were more effective than PCV13-induced antibodies against non-PCV13 serotypes 2, 22F, and 35B. Conversely, PCV13-induced antibodies showed superior binding to highly encapsulated serotype 3. It is worth noting that immunization with Gamma-PN induced comparable protection against serotype 3 during challenge despite this large disparity in Spn binding, indicating that non-CPS-specific antibodies induced by Gamma-PN are as effective as PCV13-induced CPS-specific antibodies for *in vivo* protection. Interestingly, sera from animals vaccinated with Gamma-PN+Al did not show improved binding in these assays. In fact, sera from mice vaccinated IM with Gamma-PN alone showed significantly higher binding to serotypes 22F and 35B compared to Gamma-PN+Al, despite the significant increase in total IgG for mice receiving the adjuvanted vaccine. Antibody reactivity to conserved Spn proteins was then assessed by Western blotting (Fig. 3E), with whole-cell lysates from a panel of Spn serotypes probed using pooled Gamma-PN or Gamma-PN+Al immune sera. Banding profiles were relatively similar between vaccine groups.

### Gamma-PN induces high-titer IgG with broad reactivity to Spn proteins in rabbits.

**FIG 4:**
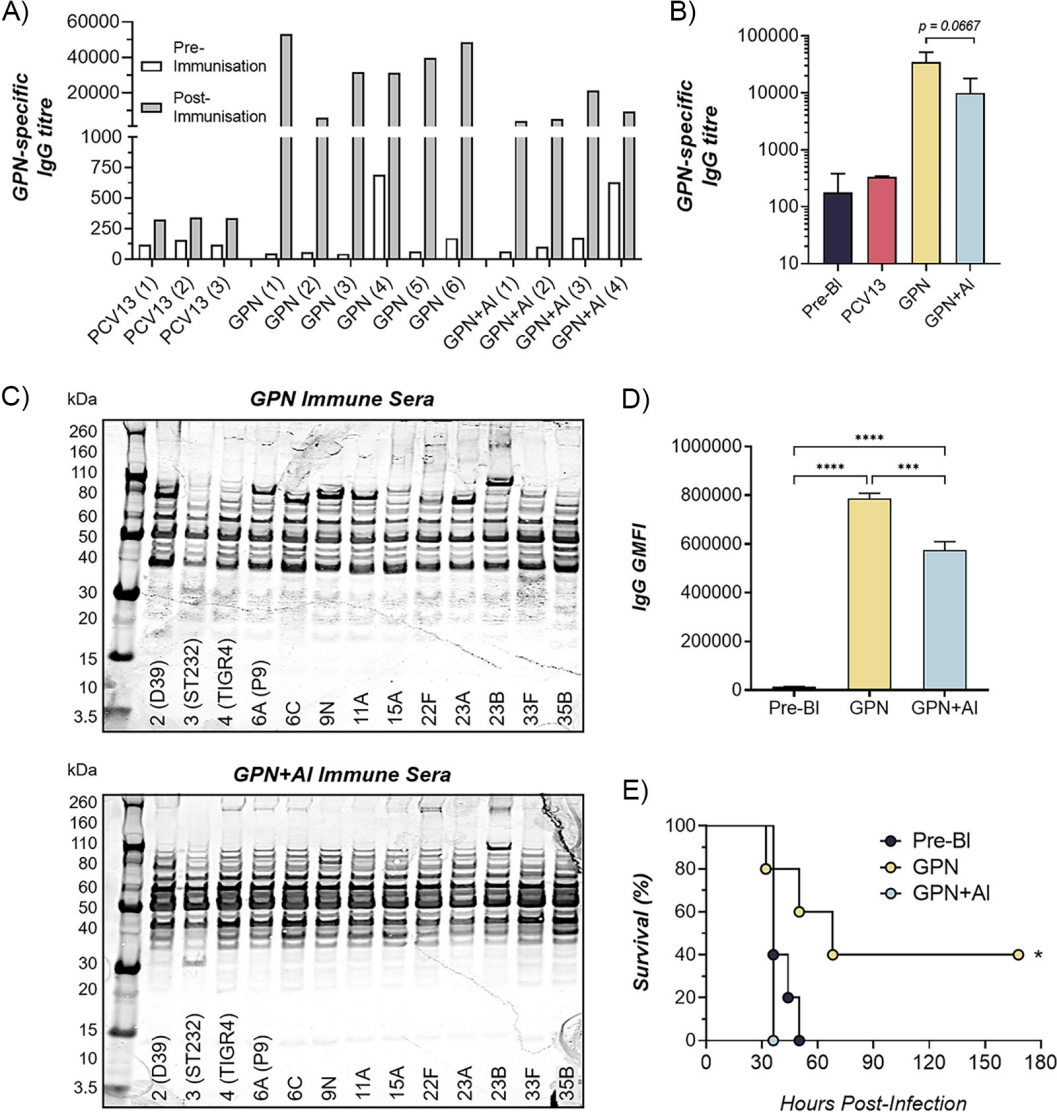
The effect of adjuvant on IgG antibody responses after Gamma-PN immunization of rabbits. Rabbits were immunized IM with PCV13, Gamma-PN, or Gamma-PN+Alhydrogel (+Al). All rabbits received three immunizations 3 weeks apart. Serum was taken from all rabbits prior to immunizations (prebleed), and again 3 weeks after the final immunization. Individual sera were tested for total IgG by ELISA, using whole-cell Gamma-PN as the capture antigen. Data presented as (A) IgG titers pre- and post-3rd immunization for each individual rabbit, and (B) mean IgG titers (± SD) within each vaccine group. IgG data were compiled from two independent experiments, and the difference between Gamma-PN groups was determined using one-way ANOVA. (C) Reactivity of pooled rabbit sera was tested by Western blot against whole-cell lysates of Spn serotypes. Western blots are representative of two independent tests of pooled immune sera. (D) Pooled sera were used to probe a microarray of purified Spn antigens printed on nitrocellulose coated glass AVID slides. Results are expressed as GMFI for total IgG binding in each group. Data analyzed by one-way ANOVA (***, *P* < 0.001; ****, *P* < 0.0001). Sera were pooled from two independent immunization experiments and tested in triplicate. (E) Survival time of mice IP-challenged with serotype 6A (10^4^ CFU/mouse) that was pretreated with pooled prebleed rabbit sera or with pooled immune sera from Gamma-PN- or Gamma-PN+Al-immunized rabbits (sera pooled from two independent immunization experiments). Survival was monitored for 7 days postchallenge, and mice were humanely euthanized when they reached a moribund state. Survival percentages analyzed by Mantel-Cox test (*, *P* < 0.05; *n* = 5 mice per group).

The observed decrease in antibody functionality and protective efficacy when Gamma-PN was adjuvanted in mice was unexpected. Thus, rabbits were utilized as a second animal model to ensure these differences were not a mouse-specific anomaly. Rabbits were IM immunized with PCV13, Gamma-PN, or Gamma-PN+Al. Sera were harvested from all animals after the 3rd immunization. Rabbits were also bled prior to immunizations, with pooled prevaccination sera used as a baseline control. ELISA was initially used to determine whether adjuvant could increase the magnitude of Gamma-PN-induced antibody responses in rabbits. Fig 4A shows that baseline titers of Spn-specific IgG in individual preimmune samples fluctuated between 125 and 600, which was expected due to the use of outbred rabbits. After Gamma-PN immunization, 10- to 100-fold increases in IgG levels were observed for all animals, with calculated titers between 10,000 and 50,000 for individual rabbits. Data also show that adding adjuvant to Gamma-PN did not significantly enhance IgG titers, instead appearing to slightly dampen total titers compared to Gamma-PN alone (Fig. 4B). As expected, PCV13-induced antibodies showed baseline reactivity against the nonencapsulated Gamma-PN vaccine that was used as coating antigen.

Pooled immune sera from each vaccine group (*n* = 2 to 4 rabbits per group) were then used to probe Western blots of whole-cell lysates of a panel of PCV13-included and nonincluded serotypes. Fig 4C shows rabbit antibodies induced by Gamma-PN had a high level of reactivity to protein antigens present in all serotypes tested. Importantly, a prominent difference in the immunodominant protein bands bound by sera from Gamma-PN and Gamma-PN+Al immunized rabbits was now observed. To gain additional information on the range of antigens bound by each immune serum type, a protein microarray of 289 purified Spn antigens was probed with Gamma-PN- or Gamma-PN+Al-immune sera. Spn proteins showing significant antibody reactivity were ranked according to mean fluorescence intensity (MFI), and the top 50 protein hits are presented in Table 1. Prebleed sera showed minimal reactivity to all 50 of these antigens. This protein array was previously used to probe antibody responses to Spn proteins in mice (12, 25), and our data show reactivity to a considerably larger number of Spn proteins for Gamma-PN-immunized rabbits compared to prior studies. Interestingly, of the 50 top antigens, 11 fell below the threshold of detection when Gamma-PN was adjuvanted, supporting the notion of impaired antibody reactivity. Comparing log_2_ fold changes between adjuvanted and nonadjuvanted Gamma-PN also showed adjuvanted sera had decreased binding to almost all the top 50 protein hits, with the exception of LivJ which showed a minor 1.2-fold increase in reactivity (Table 1). Antigens displaying >4-fold reduction in reactivity when Gamma-PN was adjuvanted were identified as: DnaK, Pbp1A, ValS, MetE, SP_1394, PrtA, PotH, AliA, and SpxB. MFI for all antigens (i.e., total IgG binding) was also quantified as a measure of total protein reactivity; Fig. 4D shows serum from Gamma-PN-immunized rabbits had significantly stronger reactivity compared with rabbits receiving Gamma-PN+Al. Finally, pooled sera from Gamma-PN-vaccinated rabbits were found to confer significant passive protection to mice against Spn serotype 6A challenge, whereas prebleed sera and sera from Gamma-PN+Al-vaccinated rabbits offered no protection (Fig. 4E), suggesting the Spn proteins showing reduced antibody reactivity were also antigenically important for *in vivo* protection.

**TABLE 1:** IgG reactivity induced by immunization with nonadjuvanted and adjuvanted Gamma-PN against antigenic Spn proteins^*a*^

Antigen details			Mean fluorescence intensity ± SD		Log_2_ fold change
TIGR4 locus tag	Gene name (if known)	Protein function	Gamma-PN (no adjuvant)	Gamma-PN+ adjuvant	Adjuvant versus no adjuvant
SP_1732	*stkP*	Serine/threonine-protein kinase StkP	42,217.8 ± 1,122.67	37,093.93 ± 722.93	−0.187
SP_1518	*mltG*	Endolytic murein transglycosylase	33,846.13 ± 812.69	32,323.27 ± 740.59	−0.066
SP_0660	*msrAB2*	Peptide methionine sulfoxide reductase MsrA/MsrB	32,985.13 ± 989.99	27,125.93 ± 568.58	−0.282
SP_0148		ABC transporter, substrate-binding protein	30,711.8 ± 2,362.48	26,194.6 ± 752.6	−0.230
SP_1604		Uncharacterized protein	29,522.13 ± 1,749.53	14,423.27 ± 2,546.63	−1.033
SP_1394		Amino acid ABC transporter, amino acid-binding protein	29,503.13 ± 1,329.58	1,355.93 ± 432.86	−4.444
SP_1527	*aliB*	Oligopeptide-binding protein AliB	27,165.47 ± 2,347.43	16,204.6 ± 3,227.17	−0.745
SP_0517	*dnaK*	Chaperone protein DnaK (HSP70)	26,577.13 ± 844.5	**19.87 ± 32.69**	−10.385
SP_0981	*prsA*	Foldase protein PrsA	26,301.47 ± 2,094.91	27,537.6 ± 927.66	0.066
SP_0453		Amino acid ABC transporter, amino acid-binding protein/permease protein	25,945.13 ± 1,861.6	21,189.93 ± 1,977.88	−0.292
SP_2169	*adcA*	Zinc-binding lipoprotein AdcA	25,479.47 ± 491.37	7,189.93 ± 965.85	−1.825
SP_1032	*piaA*	Iron-compound ABC transporter, iron compound-binding protein	24,388.13 ± 1,030.42	18,096.27 ± 1,649.89	−0.430
SP_1128	*eno*	Enolase	23,680.47 ± 1,475.8	2,4042.27 ± 1,602.22	0.022
SP_0374	*mapZ*	Midcell-anchored protein Z	23,430.8 ± 891.93	26,078.6 ± 269.49	0.154
SP_1891	*amiA*	Oligopeptide-binding protein AmiA	23,322.47 ± 3,591.01	12,425.93 ± 1,773.56	−0.908
SP_1942		Putative transcriptional regulator	23,285.13 ± 4,456.35	15,596.93 ± 2,464.72	−0.578
SP_2218	*mreC*	Cell shape-determining protein MreC	23,055.47 ± 1,714.05	13,583.93 ± 361.91	−0.763
SP_1174		Conserved domain protein	22,441.8 ± 2,228.77	12,596.6 ± 281.17	−0.833
SP_2239	*htrA*	Serine protease	22,242.13 ± 2,703.81	19,299.27 ± 1,374.49	−0.205
SP_2029	*yajC-2*	Preprotein translocase, YajC subunit	21,555.47 ± 795.13	7,709.6 ± 399.13	−1.483
SP_0568	*valS*	Valine–tRNA ligase	20,894.47 ± 865.98	**846.2 ± 793.12**	−4.626
SP_1479	*pgdA*	Peptidoglycan N-acetylglucosamine deacetylase A	20,514.8 ± 1,842.74	5,411.27 ± 557.36	−1.923
SP_0629		VanY domain-containing protein	17,791.13 ± 1,923.57	11,916.27 ± 1,183.09	−0.578
SP_0845		Lipoprotein	17,436.8 ± 1,509.69	18,895.93 ± 2,571.16	0.116
SP_0877	*fruA*	PTS system, fructose specific IIABC components	16,762.47 ± 1,323.25	12,392.27 ± 2,455.32	−0.436
SP_1368	*psr*	Psr protein	16,422.13 ± 2,437.2	20,221.27 ± 3,117.34	0.300
SP_0730	*spxB*	Pyruvate oxidase	15,773.8 ± 1,840.67	3,601.27 ± 212.76	−2.131
SP_1872	*piuA*	Iron-compound ABC transporter, iron-compound-binding protein	15,729.47 ± 473.02	18,320.27 ± 4,265.17	0.220
SP_2108	*malX*	Maltose/maltodextrin-binding protein	12,613.47 ± 1,742.34	19,832.93 ± 303.2	0.653
SP_2012	*gap*	Glyceraldehyde-3-phosphate dehydrogenase	10,625.13 ± 3,76.98	6,753.27 ± 2,76.51	−0.654
SP_1890	*amiC*	Oligopeptide transport system permease protein AmiC	9,958.47 ± 2,560.59	13,030.93 ± 1,792.25	0.388
SP_0369	*pbp1A*	Penicillin-binding protein 1A	8,312.8 ± 1701.7	**125.73 ± 217.78**	−6.047
SP_0283	*manM*	PTS system, mannose specific IIC component	8,081.13 ± 946.68	13,385.6 ± 503.76	0.728
SP_0149		Lipoprotein	8,001.47 ± 6,027.8	**8,027.6 ± 9,224.91**	0.005
SP_1650	*psaA*	Manganese ABC transporter substrate-binding lipoprotein	7,472.47 ± 2,298.1	1,904.6 ± 272.15	−1.972
SP_1388	*potH*	Spermidine/putrescine ABC transporter, permease protein	6,467.13 ± 1,744.26	1,188.6 ± 258.24	−2.444
SP_2085	*pstC*	Phosphate transport system permease protein	5,396.8 ± 2,017.12	**231.27 ± 75**	−4.544
SP_0284	*manL*	EIIAB-Man	5,374.13 ± 193.91	9,420.93 ± 723.38	0.810
SP_2223		hypothetical protein	4,849.8 ± 467.69	3,379.6 ± 354.14	−0.521
SP_0807	*ezrA*	Septation ring formation regulator EzrA	4,522.8 ± 1,316.05	**0 ± 0**	NA
SP_0659	*etrx1*	Thioredoxin family protein	4,332.8 ± 1,408.67	**3,032.6 ± 640.65**	−0.515
SP_0641	*prtA*	Serine protease, subtilase family	4,042.47 ± 451.63	**675.6 ± 242.3**	−2.581
SP_0366	*aliA*	Oligopeptide-binding protein AliA	3,950.8 ± 1,107.35	**755.93 ± 156.91**	−2.386
SP_2099	*pbp1B*	DD-transpeptidase	3,867.8 ± 445.11	1,298.93 ± 140.8	−1.574
SP_0042	*comA*	Transport/processing ATP-binding protein ComA	3,754.8 ± 3,264.97	2,590.27 ± 151.67	−0.536
SP_0719		Membrane protein, putative	3,754.47 ± 149.46	2,652.27 ± 637.07	−0.501
SP_0749	*livJ*	Branched-chain amino acid ABC transporter, amino acid-binding protein	3,063.13 ± 332.5	6,868.6 ± 588.2	1.165
SP_1400	*pstS1*	Phosphate-binding protein PstS 1	2,987.13 ± 852.5	**0 ± 0**	NA
SP_0585	*metE*	5-methyltetrahydropteroyltriglutamate–homocysteine methyltransferase	2,795.47 ± 1,408.23	**119.73 ± 160.57**	−4.545

a Antigens are sorted based on the mean fluorescence intensity (MFI) of the nonadjuvanted Gamma-PN samples. The identity of each antigen is shown by TIGR4 locus tag, gene name (if known) and protein function. The relative IgG levels are shown by the log_2_ fold change for the adjuvant versus no adjuvant Gamma-PN groups. Antigens with at least one replicate below the threshold of detection are shown in bold. Data presented as the mean fluorescence intensity ± SD of pooled serum samples from immunized rabbits (sera pooled from two independent vaccination experiments). Log_2_ fold change calculated using MFI.

### Antibodies induced by nonadjuvanted Gamma-PN are superior for encapsulated Spn binding.

**FIG 5:**
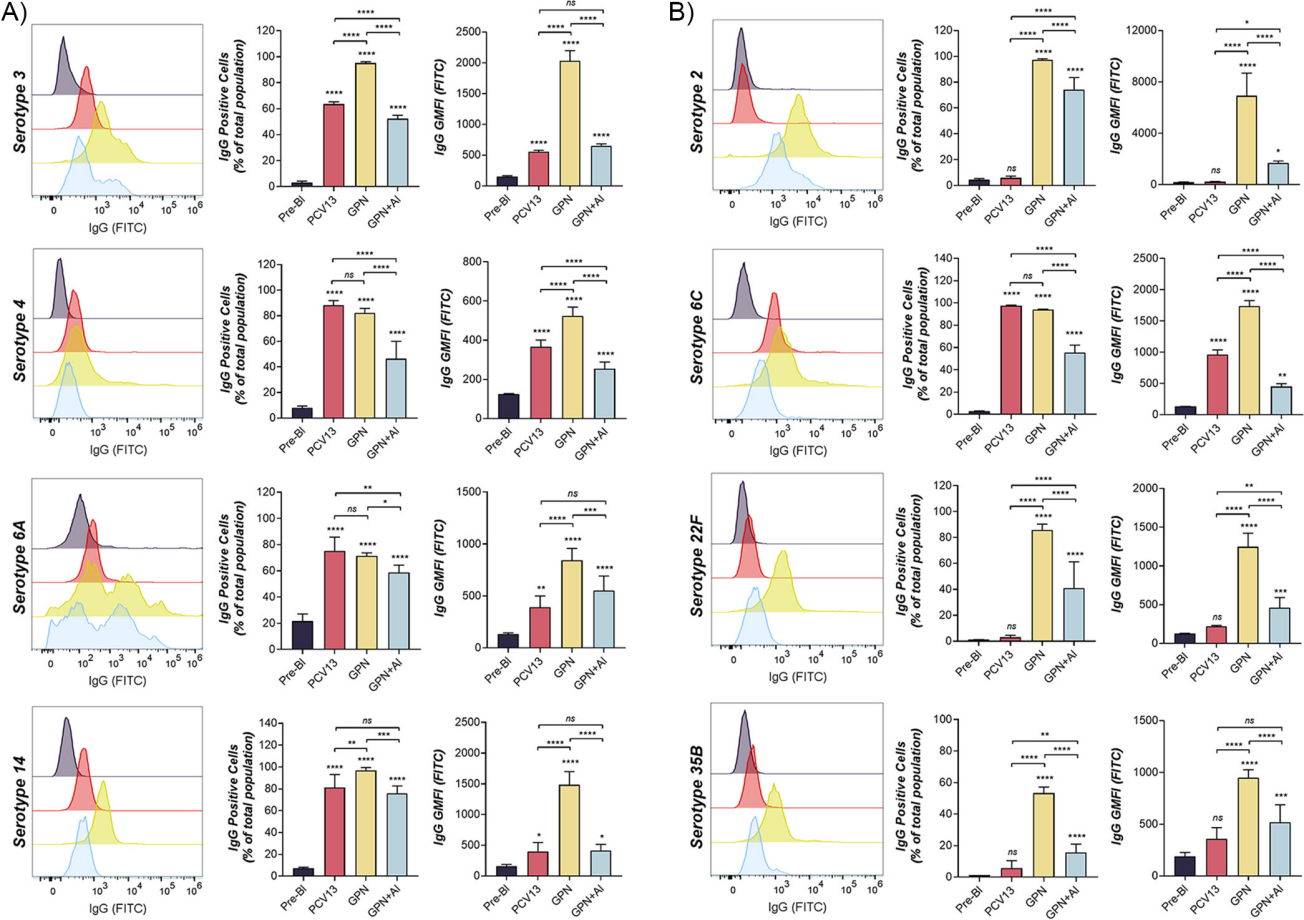
Encapsulated Spn binding by sera from Gamma-PN- and PCV13-immunized rabbits. Rabbits were immunized IM with PCV13, Gamma-PN alone, or Gamma-PN+Al (0.5 mg Gamma-PN/dose in each case). All rabbits received three immunizations 3 weeks apart. Serum was taken from all rabbits 3 weeks after the final immunization and pooled within each immunization group (*n* = 2 to 4 rabbits per group per experiment). Pooled sera were tested for IgG binding to encapsulated Spn using flow cytometry. Serum was also harvested from all rabbits prior to any immunizations and pooled in equal volumes as a baseline control for nonspecific binding (termed prebleed [Pre-Bl], with 8 rabbit samples total pooled per experiment). Pooled immune sera and prebleed samples were then tested in technical triplicate for flow cytometry experiments against (A) PCV13-included serotypes, or (B) non-PCV13 serotypes. Histograms are representative of two independent immunization experiments, with subsequent analysis by independent flow cytometry experiments. Data for GMFI and percentage of positive cells are compiled from both independent sets of experiments, and are presented as mean ± SD. Quantitative data were analyzed by one-way ANOVA (significance compared to prebleed control is indicated on top of each column, and significance between vaccine groups is indicated by horizontal lines; ***, *P* < 0.0001; ***, *P* < 0.001; **, *P* < 0.01; *, *P* < 0.05; ns, not significant).

Binding assays were again performed to compare the performance of protein-specific antibodies induced by Gamma-PN to the CPS-specific antibodies induced by PCV13. Live Spn serotypes were incubated with pooled rabbit immune sera, then probed with a fluorophore-conjugated anti-IgG antibody for analysis by flow-cytometry. Fig. 5 shows rabbit antibodies induced by the nonencapsulated Gamma-PN were capable of recognizing protein targets on the surface of all capsular Spn serotypes tested. Note that high binding of capsule-specific antibodies present in PCV13-induced sera confirmed that capsule was present on the relevant Spn strains tested. Antibodies induced by Gamma-PN alone were equivalent or superior to PCV13-induced antibodies for binding to all tested serotypes, including the highly encapsulated serotype 3. This differs to data observed for murine antibody binding to serotype 3 (Fig. 3D), suggesting differences in species-specific vaccine processing and highlighting the importance of testing multiple animal models for vaccine efficacy. Immune sera from PCV13-vaccinated rabbits did show binding to the nonincluded serotype 6C, consistent with previously reported cross-reactivity among Spn serogroup 6 (26–28). Regardless, Gamma-PN-induced sera showed significantly higher FITC geometric mean fluorescence intensity (GMFI) than PCV13-induced sera when reacting with 6C, indicating a higher density of antibodies were bound to individual Spn cells. Furthermore, these data demonstrate stark differences in antibody functionality between the Gamma-PN and Gamma-PN+Al groups. FITC GMFI and total percentages of IgG-bound cells were both significantly lower when probing Spn with sera from rabbits receiving adjuvanted Gamma-PN compared to the nonadjuvanted counterpart. This occurred for every Spn serotype tested, and unequivocally demonstrates that antibody reactivity was negatively affected by formulation of this WCV with adjuvant.

### Nonadjuvanted Gamma-PN induces OPKA responses against PCV13-included and nonincluded serotypes.

Vaccine performance was next compared using the opsonophagocytic killing assay (OPKA), which has been standardized using differentiated HL-60 cells (29–32) and is considered a gold-standard assay to test the potential clinical efficacy of new Spn vaccines. Individual rabbit sera (taken after the final immunization with Gamma-PN, Gamma-PN+Al, or PCV13) were tested using the established protocol from the Bacterial Respiratory Pathogen Reference Laboratory, UAB. Background Spn killing mediated by pooled prebleed sera was also tested. The Opsotiter 3 program (kindly provided by Robert L. Burton, UAB) was used to calculate the precise reciprocal of serum dilution where 50% killing of the input CFU titer was reached, also termed the opsonic index (OI). Geometric mean OI values for each vaccine group (*n* = 3 to 6 rabbits per group) are listed in Table 2. These data show Gamma-PN with no adjuvant was comparable or superior to PCV13 in mediating opsonophagocytic killing against PCV13-included serotypes 6A and 23F, and more effective than PCV13 against nonincluded serotypes 9N, 11A, 15A, 22F, 23B, 33F, and 35B. Additionally, mixing Gamma-PN with adjuvant reduced opsonophagocytic killing against most of these tested serotypes.

**TABLE 2:**
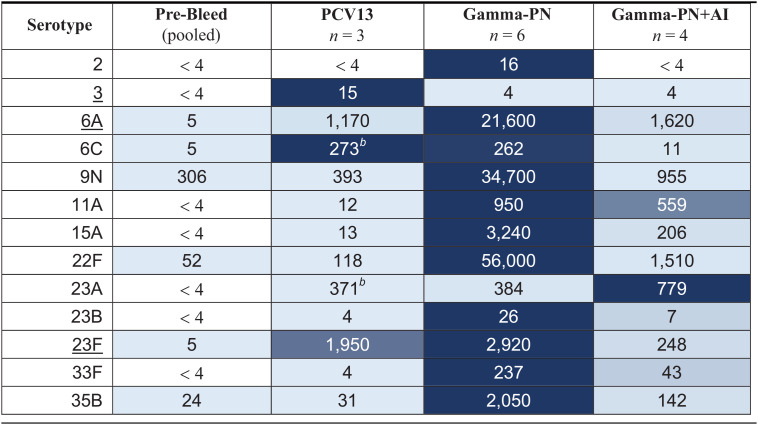
Geometric mean OI for rabbits immunized IM with PCV13, Gamma-PN, or Gamma-PN+Al^*a*^

a Individual immune sera (*n* = 3–6 rabbits per vaccine group) were tested by the opsonophagocytic killing assay (OPKA) with differentiated HL-60 cells against PCV13-included (underlined) and nonincluded Spn serotypes. The Opsotiter3 program was used to calculate opsonic index (OI) values for individual serum samples, and the geometric mean OI (GMOI) for each vaccine group is presented here. Data show the GMOI pooled from two independent experimental repeats. Prebleed sera pooled from all rabbits was used as a baseline control for nonspecific killing. A value of “<4” indicates no killing activity was detected in the OPKA even with neat sera (final dilution of 1/4 when mixed with other assay components). Table cells are colored based on a gradient from the highest (dark blue) to lowest (light blue) GMOI measured for each individual serotype.

b Cross-reactivity of PCV13 against the nonincluded serotypes 6C and 23A has been reported in the literature.

**FIG 6:**
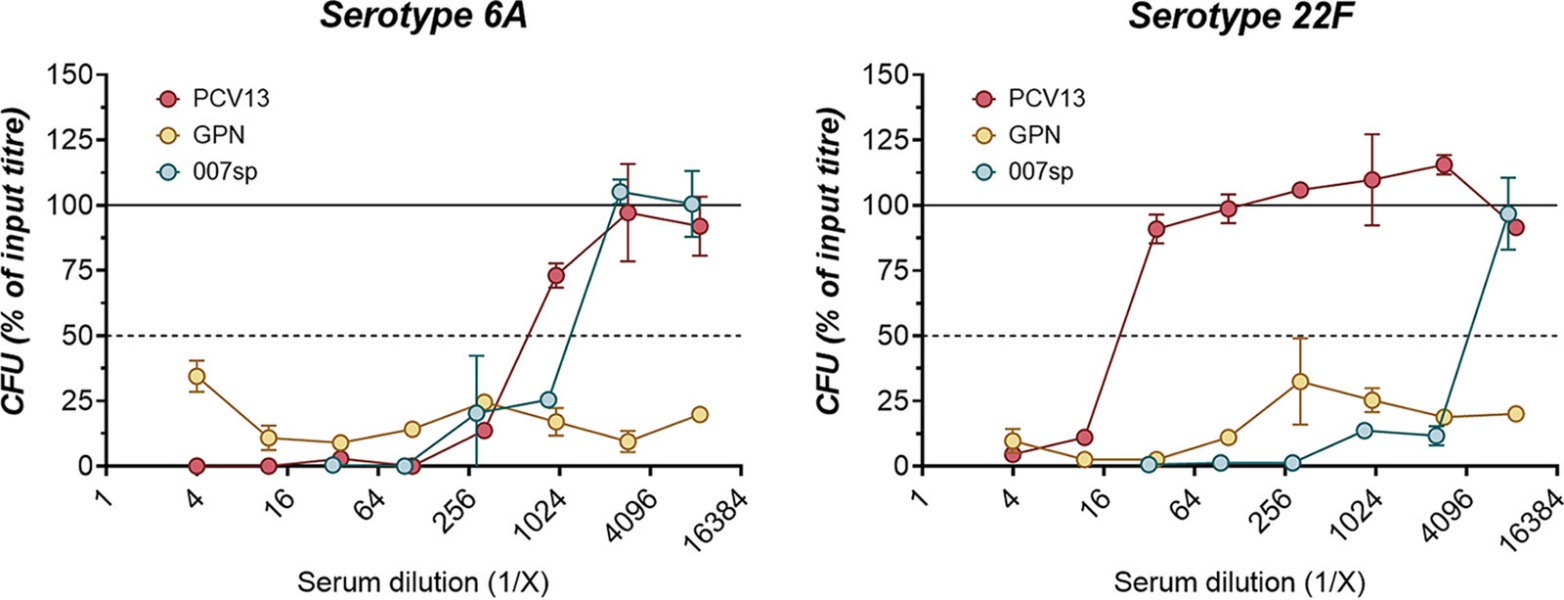
External validation that nonadjuvanted Gamma-PN induces superior OPKA responses against a PCV13-included (6A) and nonincluded serotype (22F). Rabbits were immunized IM with PCV13 or nonadjuvanted Gamma-PN (GPN). All rabbits received 3 immunizations 3 weeks apart, and sera were harvested 3 weeks after the final immunization. Pooled immune serum samples (*n* = 3 for PCV13, *n* = 6 for Gamma-PN) were titrated using OPKA by a third-party laboratory. Pooled human reference sera from the Pneumovax23 clinical trial was also tested (007sp). Titration curves show the percentage of input CFU recovered after OPKA with serially diluted sera. The dashed line in each panel represents 50% recovery of the input CFU (i.e., 50% killing), and the solid line indicates 100% recovery (i.e., 0% killing) relative to no sera controls. Pooled sera were tested in technical duplicate, and individual data points indicate the mean ± SD of CFU recovered at each serum dilution. Data were generated by external laboratory New Vaccines, MCRI. Data indicate results of a single OPKA experiment.

OPKA results were externally validated by a third-party laboratory (New Vaccines Laboratory, Murdoch Children’s Research Institute). OPKA data were generated for serotype 6A (PCV13-included) and 22F (nonincluded) and are shown in Fig. 6. These data confirm nonadjuvanted Gamma-PN was significantly more effective in mediating opsonophagocytic killing of encapsulated Spn for a PCV13-included and a nonincluded serotype. Gamma-PN was able to mediate >90% killing of input CFU across all tested serum dilutions, and yielded OI values >8,748 against serotypes 6A and 22F. Comparatively, PCV13 returned OI values of 622 and 19 in the same assay. Pooled human reference sera from 287 participants receiving Pneumovax 23 (007sp) was also included in these OPKA validations and showed high levels of killing against the 6A and 22F serotypes. This was expected as serotype 22F is included in the composition of Pneumovax 23, and while 6A is not explicitly included, the closely related 6B is present and cross-reactivity between serogroup 6 has been reported previously. Human subjects may have also been naturally exposed to 6A prior to sera collection, with IgG analysis showing the presence of anti-6A IgG within 007sp (33). Despite high reactivity of the 007sp human reference sera, Gamma-PN-immune sera were superior in these external assays.

## DISCUSSION

Spn is a major human pathogen that continues to cause >1 million deaths annually, particularly in young children and the elderly. Current vaccines cover a limited range of Spn serotypes and are unable to protect against emerging nonvaccine serotypes and unencapsulated isolates. This has led to serotype replacement in multiple regions (4–6) with many antibiotic-resistant nonvaccine serotypes (7–10). Hence, there is an urgent need for new strategies to induce serotype-independent immunity that are also globally accessible.

This study details the efficacy of Gamma-PN as a serotype-independent Spn WCV and demonstrates noninferiority to PCV13 for protection against challenge (Fig. 1), binding to encapsulated Spn (Fig. 5), and OPKA responses (Fig. 6, Table 2) against PCV13-included and nonincluded serotypes. Additionally, adjuvant was not required for induction of significant protection by Gamma-PN against tested Spn isolates (heavily encapsulated serotype 3, serotype 6A, and a human isolate of 22F), and inclusion of adjuvant actively reduced Gamma-PN-induced antibody functionality in rabbits and mice. The effect was most pronounced in rabbits, where Alhydrogel failed to increase IgG titers in serum, and significantly decreased antibody functionality when assessed by protein microarray (Fig. 4D, Table 1), passive protection assay (Fig. 4E), Spn binding assays (Fig. 5), and OPKA (Table 2). Adding adjuvant to Gamma-PN also increased local inflammation at the immunization site (Fig. 2), with no apparent benefit to vaccine performance.

This finding distinguishes Gamma-PN from Spn vaccines developed by other groups. For example, Malley and colleagues (16, 17, 34) developed a similar Spn vaccine (RM200) and have reported phase 2 trials (ClinicalTrials.gov Identifier: NCT02097472). RM200 is chemically inactivated, and murine studies showed protective efficacy relied on administration with cholera toxin-based adjuvants (16, 17), or aluminum hydroxide as adjuvant (34). Meta-analysis highlighted that the majority of other whole-inactivated Spn vaccines in the literature also require coadministration with adjuvant for protective efficacy (35).

A small number of adjuvants are currently licensed for human use, with aluminum hydroxide (e.g., Alhydrogel [AH]) and aluminum phosphate (e.g., AdjuPhos [AP]) being the most commonly used (36), including in the commercially available PCV13. Alhydrogel was selected for this study as a representative aluminum-based adjuvant. Both AH and AP have large adsorptive surfaces, with adsorption of antigens intended to increase retention at the injection site. Aluminum-based adjuvants are also associated with recruitment of macrophages, dendritic cells, neutrophils, and eosinophils to the injection site in a time-dependent manner (37) and a Th2 bias with a marked increase in IL-4-secreting CD4^+^T cells (38). Consistent with this, we observed a bias toward Th2-type IgG subclasses here when Gamma-PN was adjuvanted (Fig. 3). Importantly, this also caused unexpected detrimental effects on antibody functionality and protective efficacy.

Strong adsorption of soluble antigens to AH particles is known to increase antigen uptake by DC’s (39), though overly strong interactions may negatively affect the structural stability of antigens and interfere with epitope availability for proteolytic processing and binding by MHC-II molecules (40, 41). High adsorptive capacity of AH is associated with poor antigen elution and can interfere with antibody production and T-cell activation in response to injection of soluble proteins (42). Additionally, Hogenesch et al. (43) demonstrated AH has a high adsorptive capacity for whole-cell Spn. Authors postulated that strong antigen adsorption may be less detrimental to immune responses against whole bacteria, as this antigen type is ~1 μm in diameter while adjuvant particles are <50 nm (44). However, high-affinity adsorption of AH to soluble or membrane-bound Spn proteins is likely to have detrimental effects on antigen processing, potentially masking epitopes from immune recognition and interfering with MHC-II presentation. In line with this, reduced antibody reactivity to many Spn proteins was observed when Alhydrogel was used to adjuvant Gamma-PN (Table 1).

The surface charge of AH is also positive at neutral pH, resulting in differing affinities for antigens that adsorb via electrostatic interactions (44). Spn is typically considered to have a net negative charge due to capsular polysaccharides (45, 46), with cell-surface structures (e.g., choline-binding proteins) also contributing (47). Purified Spn proteins pneumococcal surface antigen A (PsaA) and serine-threonine protein kinase (StkP) adsorb to a greater degree to AH than to AP, indicating negative charge of these soluble proteins (48). Whole-cell Rx1 is also reported to have a similar electronegative charge to the parent strain D39 (47), suggesting removal of capsule does not dramatically alter the net charge of serotype 2 Spn. It can therefore be inferred that the unencapsulated Gamma-PN (also derived from the D39 descendant Rx1) would be highly negatively charged and would readily adsorb to positively charged AH.

Antigens additionally adsorb to aluminum adjuvants via ligand exchange, where surface hydroxyls are replaced by terminal phosphate groups of phosphorylated antigens, creating a covalent bond. AH has many surface hydroxyls and thus has very high affinity for phosphorylated antigens (44). Viral vaccine studies have reported correlations between increased adsorption of phosphorylated proteins and decreased immunogenicity (42, 49, 50). Spn proteins PcpA, PhtD, and Ply strongly bind AH due to ligand exchange, with AH adsorption being additionally associated with decreased protein stability during long-term incubation (51). Thus, excessively high binding of whole-cell Spn and/or soluble protein components by AH adjuvant is likely to occur via both electrostatic interaction and ligand exchange, and the aforementioned studies link this high adsorption to reduced vaccine immunogenicity.

The negative influence of Alhydrogel on Gamma-PN-induced antibodies could additionally be related to a biased IgG subclass profile. While the propensity of adjuvants to bias immunity along a Th2-type pathway is recognized to enhance total antibody production, the effect on antibody functionality must be given equal consideration. For Gamma-PN+Al-immunized mice, IgG1 was the predominant subclass, indicative of a classic Th2-type response. Conversely, IgG1 was more proportionate to other subclasses for mice immunized with Gamma-PN alone (Table S3), indicating a more balanced Th1/Th2-type response. IgG1 antibodies can neutralize toxins and viruses through steric hindrance, but they are reported to be ineffective in complement fixation and for triggering FcγR-mediated effector functions. In contrast, IgG2a and IgG2b are substantially better at fixing complement, and for Fc receptor binding and associated phagocytic functions (52–56). A broader Th1-skewed subclass distribution would therefore be beneficial against Spn, and potential saturation with the suboptimal IgG1 seen for Gamma-PN+Al-immunized mice could explain disparities in antibody titer and protective efficacy.

Detrimental effects of Th2-skewed responses have been reported before. Jang et al. (14) developed an Spn vaccine comprised of TIGR4 deficient in lipoprotein diacylglyceryl transferase (*lgt*) and subsequently inactivated with gamma irradiation, similar to Gamma-PN. However, the killed TIGR4 vaccine induced less effective protection against Spn challenge and less IFN-γ production by splenocytes compared to the live Δ*lgt* Spn vaccine counterpart, which was attributed to the vaccine inactivation method shifting the IgG subclass profile to be IgG1-dominated (14). It is worth noting the TIGR4 vaccine was treated with 5 kGy of gamma irradiation at room temperature, which may not be sufficient to reach a sterility assurance level of 10^−6^, whereas Gamma-PN is irradiated with higher doses while frozen on dry-ice for better maintenance of antigen integrity (57). Investigation of a group A Streptococcus vaccine also showed Alhydrogel induced Th2-type IgG subclass profiles, and negatively affected protection rates compared to other adjuvants (58). Note that no comparison was made to the vaccine alone, though adjuvants associated with better protection also induced more balanced Th1/Th2 responses.

Importantly, other killed whole-cell Spn preparations have been reported to induce minimal OPKA responses in animals after IN (19) or SC immunization (59), and the RM200 vaccine did not produce detectable OPKA responses in humans after adjuvanted vaccination (60). Here, OPKA responses for nonadjuvanted Gamma-PN were similar in efficacy to PCV13-induced responses against PCV13-included serotypes, with Gamma-PN also outperforming PCV13 against the included serotype 6A. Nonadjuvanted Gamma-PN was then superior to PCV13 for opsonophagocytic killing against 8 different non-PCV13 serotypes, including the emergent serotypes 22F, 33F, and 35B. Higher OPKA efficacy of Gamma-PN compared to PCV13 against serotypes 6A and 22F was also externally validated (Fig. 6). As Gamma-PN lacks CPS, the antibodies mediating OPKA responses are presumably largely protein-specific. Experimental pneumococcal vaccines comprising various purified protein antigens have been under study for decades, but to our knowledge, none of these have been shown to elicit a significant OPKA response. The likely explanation for the strong OPKA responses observed in the present study is the breadth and strength of the antiprotein response elicited by Gamma-PN, as shown in Table 1. This enables a much larger total amount of IgG to be bound to the surface of encapsulated pneumococci than can be achieved when just a few protein species are targeted. Indeed, our flow cytometry data (Fig. 5) showed that total IgG binding using Gamma-PN serum matched or exceeded binding achieved using the CPS-specific PCV13 serum for several vaccine-included serotypes. A further point of significance is that unlike CPS-specific antibodies elicited by PCV13, the antiprotein responses elicited by Gamma-PN have the potential to neutralize the biological activities of many of their protein targets. Blockade of surface protein function, which may be critical for pathogenesis, may have contributed to the strong protection against challenge with type 3 Spn seen in Fig. 1, despite a weaker OPKA response relative to PCV13 for this particular serotype.

Overall, this study demonstrates the efficacy and clinical suitability of the WCV Gamma-PN. To our knowledge, no study has demonstrated superior efficacy of a nonadjuvanted whole-inactivated Spn vaccine, either when tested against its own adjuvanted counterpart or against the licensed PCV13. We conclude Gamma-PN is well-suited for clinical evaluation without the need for an adjuvant, which brings many cost and tolerability benefits in addition to the immune-specific benefits observed here.

## MATERIALS AND METHODS

### Ethics statement.

Animal experimentation was conducted in strict accordance with the Australian Code of Practice for Care and Use of Animals for Scientific Purposes (7th edition [2004] and 8th edition [2013]) and the South Australian Animal Welfare Act 1985. Murine experimental protocols were approved by the Animal Ethics Committee at The University of Adelaide (Approval number S-2016-183). Rabbit experimentation was conducted at South Australian Health & Medical Research Institute Preclinical Imaging & Research Laboratories (SAHMRI-PIRL) at Gilles Plains, South Australia. Rabbit experimental protocols were approved by the SAHMRI Animal Ethics Committee (approval numbers SAM 399.19 and SAM-20-038).

### Vaccine strain.

The strain was based on the nonencapsulated Rx1 strain previously modified to delete autolysin (*lytA*) and attenuate pneumolysin (*ply*) genes (detailed in Babb et al. [18]), and to delete the pneumococcal surface antigen A (*psaA*) gene (detailed in David et al. [20]). The resulting vaccine strain Rx1 (ΔLytA, PdT, ΔPsaA) is referred to as ‘GPN-002’ in this study. A virulence of GPN-002 was confirmed by intraperitoneal (IP) challenge of female Swiss mice at 7 weeks of age, purchased from University of Adelaide Laboratory Animal Services (LAS) facility and housed at the LAS facility for a week to acclimate. Mice were injected IP with live serotype 2 strain D39 (1 × 10^4^ CFU/mouse in phosphate-buffered saline [PBS]) or live GPN-002 (1 × 10^8^ CFU/mouse in PBS). Mice were monitored multiple times daily for development of clinical symptoms. Mice were euthanized when they became moribund in accordance with ethics approval S-2016-183. Weights were recorded daily for all mice for 2 weeks postchallenge (Fig. S1A).

10.1128/mbio.02367-22.1 FIG S1 Avirulence of the live GPN-002 vaccine strain, and inactivation by gamma-irradiation. (A) Female mice at 7 weeks of age were injected IP with live serotype 2, strain D39 (at 5 × 10^4^ CFU/mL in PBS, 200 μL injected per mouse) or the live GPN-002 vaccine strain (at 5 × 10^8^ CFU/mL in PBS, 200 μL injected per mouse). All mice were monitored multiple times daily for development of clinical symptoms and were euthanized when mice became moribund. Data are presented as mean survival time (top panel) and as weight change for individual mice (bottom panel) (*n* = 5 mice per group). Survival time data analyzed by Mann-Whitney U-test (**, *P < *0.01). (B) Concentrated GPN-002 stocks at 5 × 10^9^ CFU/mL were inactivated with increasing doses of gamma-irradiation from a Co^60^ source at ANSTO in Lucas Heights NSW, Australia. Samples were kept frozen on dry-ice for the entirety of irradiation treatment, and during storage and transport between facilities. Samples were plated on blood agar plates for D_10_ analysis to confirm the inactivation of this vaccine strain. Data presented as mean CFU/mL (± SD, error bars are hidden by the symbols due to small size) of samples plated in duplicate. Download FIG S1, TIF file, 2.0 MB. Copyright © 2022 David et al. 2022 David et al. https://creativecommons.org/licenses/by/4.0/ This content is distributed under the terms of the Creative Commons Attribution 4.0 International license.

### Vaccine manufacture and inactivation.

GPN-002 vaccine strain was statically grown in soytone media (Table S2). GPN-002 was first inoculated into soytone media to optical density at 600 nm (OD_600_) = 0.05 in 50 mL final volume, and grown to OD_600_ = 0.5 at 37°C. This primary culture was inoculated into 450 mL of soytone to OD_600_ = 0.05 and grown again to OD_600_ = 0.5. 500 mL culture then subcultured into 4.5 L of soytone and grown to a final OD_600_ of 1.7. Cultures were immediately cooled on ice and centrifuged at 12,320 × *g* at 4°C. Cultures were washed twice in PBS, resuspended to a final concentration of 5 × 10^9^ CFU/mL in PBS + 13% glycerol. Concentrated GPN-002 stocks were frozen at –80°C in 1.5 mL aliquots.

10.1128/mbio.02367-22.7 TABLE S2 Detailed composition of soytone medium (prepared at pH 7.5). Download Table S2, DOCX file, 0.01 MB. Copyright © 2022 David et al. 2022 David et al. https://creativecommons.org/licenses/by/4.0/ This content is distributed under the terms of the Creative Commons Attribution 4.0 International license.

Frozen GPN-002 was inactivated with 16 kGy of gamma irradiation from a Cobalt-60 source at the Australian Nuclear Science and Technology Organization (ANSTO), Lucas Heights NSW, Australia. Samples were kept frozen on dry ice for the full irradiation treatment, and during storage and transport between facilities. Irradiation dose was selected based on prior work (20). D_10_ analysis (Fig. S1B) confirmed inactivation of GPN-002 from doses of 9 kGy onwards. To determine protein content, 16 kGy gamma-irradiated GPN-002 (referred to herein as Gamma-PN) was lysed with 1% sodium dodecyl sulfate (SDS) at 100°C for 5 min, and total protein quantified at 7.90 mg/mL using the DC protein assay kit (Bio-Rad, catalogue no. 5000112) as per manufacturer’s instructions.

### Mouse immunizations.

Female Swiss mice at 4 to 5 weeks of age were purchased from ARC Animal Facility (WA, Australia), or University of Adelaide Laboratory Animal Services (LAS), then housed at the LAS facility in Adelaide for a week to acclimatize. For intramuscular (IM) immunizations, mice received 25 or 50 μg total protein (depending on experimental requirements) of irradiated Gamma-PN vaccine in 50 μL PBS or in 50 μL PBS+Alhydrogel. Control mice received 50 μL of plain PBS, 50 μL of PBS+Alhydrogel, or the commercially available Prevnar13 (PCV13) vaccine (50 μL PCV13 per dose). Alhydrogel (aluminum hydroxide adjuvant, Invivogen catalogue no. vac-alu-250) was used for the entirety of this study at 10% final concentration (vol/vol). All IM vaccines were administered in the rear quadricep muscle, with each mouse receiving 3 vaccinations in total at 2-week intervals. Serum was harvested from all animals by submandibular bleeding 1 week after final immunizations. For assessment of acute reactogenicity, mice were IM immunized once in each quadricep muscle prior to tissue harvest 6 to 72 h later.

For intranasal (IN) vaccinations, animals were first anaesthetized by IP injection of ketamine anesthetic (10% ketamine + 1% xylazil vol/vol) suspended in injection-grade H_2_O, dosed at 10 μL per gram of body weight. Once anaesthetized, 25 μg total protein of Gamma-PN suspended in 30 μL of PBS (no Alhydrogel) was administered through the nostrils. Mice were individually monitored to ensure regular breathing and recovery from anesthetic. IN vaccinations were administered a total of 3 times at 2-week intervals, and serum was harvested from all animals by submandibular bleeding 1 week after final immunizations.

### Rabbit immunizations.

Outbred New Zealand white rabbits were purchased from Flinders University SA or Pipers Farm NSW, and housed at SAHMRI-PIRL in Adelaide. Male and female rabbits purchased at 12 to 16 weeks of age were allowed 1 week to acclimatize to housing prior to immunizations. Rabbits were immunized in the hind flank with Gamma-PN (0.5 mg Gamma-PN in 0.5 mL PBS), Gamma-PN+Alhydrogel (0.5 mg Gamma-PN in 0.5 mL PBS with 10% Alhydrogel vol/vol), or PCV13 (0.5 mL PCV13/dose, equivalent to a human dose). Rabbits received 3 immunizations in total at 3-week intervals. Blood collection was performed from the ear vein or saphenous vein to obtain serum 2 days prior to immunizations (termed prebleed), and 10 days after final immunization. Immunizations were conducted on two independent batches of rabbits (treated approximately 6 months apart) as part of this study.

### Spn-specific ELISA.

First, 96-well Greiner Hi-Bind plates were incubated overnight with whole-cell GPN-002 as coating antigen (5 × 10^6^ CFU/well in 100 mM bicarbonate/carbonate buffer, pH 9.6). After washing and blocking of the remaining sites with 2% skim milk, serially diluted sera were added to wells and incubated for 2 h. Spn-specific IgG and subclasses IgG1, IgG2a, IgG2b, and IgG3 were detected with the following antibodies diluted in blocking buffer: HRP goat antimouse IgG (H+L) (Thermo-Fisher, 1/10,000 dilution), AP goat antirabbit IgG (H+L) (Bio-Rad, 1/10,000), HRP rabbit antimouse IgG1 (Zymed, no. 61-0120, 1/1,000), HRP rabbit antimouse IgG2a (Zymed, no. 61-0220, 1/1,000), HRP rabbit antimouse IgG2b (Invitrogen, no. 610320, 1/1,000), and HRP goat antimouse IgG3 (Southern Biotech, no. 1100-05, 1/4,000). Color was developed using a BD OptEIA TMB substrate kit (BD, no. 555214) for HRP conjugates, and pNPP disodium hexahydrate tablets (Sigma, no. N9389) for AP conjugates. End-point titers were calculated by fitting nonlinear regression model “variable slope [agonist] versus response, four parameters” to log_10_ transformed data, and determining the EC_50_ value for individual samples.

### IgG binding to different Spn serotypes.

Wild-type (encapsulated) Spn isolates were grown to OD_600_ = 0.2 in Todd Hewitt broth + 0.5% yeast extract (THY). Cultures were washed 1× in PBS by centrifuging at 3,273 × *g* for 10 min, then resuspended in PBS + 13% glycerol at OD_600_ = 2.0 and frozen at –80°C until use. Strains were resuspended at 1 × 10^8^ CFU/mL in PBS immediately prior to use in binding assays. Next, 100 μL of bacteria were added to wells of a U-bottom 96-well tray, spun at 3,273 × *g* for 10 min to pellet, and washed 2× with PBS. Pellets were resuspended in 50 μL of murine or rabbit sera diluted 1/200 in PBS + 1% bovine serum albumin (BSA). Serum samples were pooled in equal volumes within each vaccine group and heat-inactivated at 56°C for 30 min prior to use. Pellets were incubated in primary sera for 45 min on ice, then washed 3 × in PBS. Pellets were resuspended in 50 μL secondary antibody in PBS + 1% BSA, using Alexa-Fluor488-conjugated goat antimouse IgG (H+L) (Invitrogen, catalog no. A-1101, 1/500 dilution) or Alexa-Fluor488-conjugated donkey anti-rabbit IgG (Thermo Fisher, catalog no. A-21206; 1/1,000 dilution) to detect murine and rabbit primary sera, respectively. After 45 min on ice, pellets were washed 3 × in PBS and resuspended in 200 μL 2% paraformaldehyde (PFA; 0.2-μm filter sterilized) for acquisition using an Accuri Flow cytometer; 10,000 events were acquired per sample. PFA alone was used as a blank control to ensure the absence of non-Spn events (<100 events in 50 μL of PFA-only control was required for each experiment to be considered valid). Gating strategy for IgG-positive events is shown in Fig. S2.

10.1128/mbio.02367-22.2 FIG S2 Example of gating Strategy for IgG positive Spn bacteria by flow cytometry. Spn serotype 2 strain D39 at 1 × 10^8^ CFU/mL in PBS was incubated in polyclonal rabbit sera diluted 1/200 in PBS + 1% BSA. Serum samples were pooled in equal volumes within each vaccine group (PCV13-vaccinated or Gamma-PN-vaccinated) and heat-inactivated at 56°C for 30 minutes prior to use. Prebleed sera was taken from all rabbits prior to immunizations and similarly pooled + heat-inactivated before use in the assay. Control Spn samples were incubated in PBS + 1% BSA only (No sera control). After serum incubation, bacteria were washed and incubated in AlexaFluor488-conjugated anti-IgG. Bacteria were washed in PBS again and resuspended in 2% PFA (0.2 μm filter sterilized) for acquisition on an Accuri flow cytometer. 10,000 events were acquired per sample. Representative plots are shown to for the unstained Spn control, and Spn incubated with rabbit sera. Plots illustrate the typical size distribution of Spn on the Accuri flow cytometer, and the placing of the IgG positive gate relative to the unstained control samples. This representative gating strategy was used for all IgG binding experiments in this study. Download FIG S2, TIF file, 2.3 MB. Copyright © 2022 David et al. 2022 David et al. https://creativecommons.org/licenses/by/4.0/ This content is distributed under the terms of the Creative Commons Attribution 4.0 International license.

### Western blots.

Wild-type Spn isolates were grown as above and quantified using the DC protein assay kit prior to SDS-PAGE. Samples were diluted in milliQ water + 1× LUG buffer and boiled at 100°C for 5 min. Whole-cell lysates were loaded into duplicate wells on precast NuPAGE 4 to 12% Bis-Tris protein gels (Invitrogen, catalog no. NW04127) at 3.75 μg protein/well. Proteins were transferred to nitrocellulose membranes using the iBlot gel transfer system (Invitrogen, catalog no. IB301002). Membranes were blocked in 2% skim milk buffer, sera from immunized or control animals were pooled and heat inactivated as described for the IgG binding assay, then diluted 1/1,000 (mouse) or 1/5,000 (rabbit) in 2% skim milk blocking buffer and used to probe membranes. After overnight incubation at room temperature (RT) with orbital shaking, membranes were washed, and bound primary antibody was detected with Odyssey IRDye-800CW goat antimouse IgG (no. 926-32210) or Odyssey IRDye-680RD goat antirabbit IgG (no. 926-68073) for 1 h with shaking at RT. Membranes were washed in milliQ water prior to imaging with the LI-COR Odyssey Imaging system.

### Acute reactogenicity analysis.

Mice were immunized once in both quadricep muscles with Gamma-PN, Gamma-PN+Alhydrogel, or PCV13 as described in Animal Immunizations. Control mice were mock immunized with PBS or PBS+Alhydrogel or received no treatment to indicate baseline status. Baseline controls were euthanized immediately, and both quadricep muscles were excised. Tissues from treated animals were similarly harvested 6 to 72 h postimmunization, and each quadricep muscle was processed immediately after harvest for cytokine analysis or histology. The right quadricep muscle from each animal was rinsed in PBS and placed in bead tube with 1 mL PBS + protease inhibitor cocktail (Sigma, no. P8340). Tissues were homogenized for 3 × 30 s cycles at 5,000 rpm, then transferred to fresh 2-mL Eppendorf tubes and spun at 13,523 × *g* for 20 min at 4°C. Supernatants were transferred to a new tube and stored at −80°C until analysis. Muscle supernatant samples were analyzed by a multiplex murine cytokine and chemokine panel (Crux Biolab, Melbourne Australia) to detect G-CSF (CSF-3), GM-CSF, IL-1 beta, MCP-1 (CCL2), MCP-3 (CCL7), MIP-1 alpha (CCL3), MIP-1 beta (CCL4), and MIP-2 alpha (CXCL2). The left quadricep muscle from each animal was rinsed in PBS and placed in 20 mL of 10% neutral buffered formalin (100 mL 37% formaldehyde, 900 mL distilled water, 4 g monobasic sodium phosphate [NaH_2_POH_2_O], 6.5 g dibasic sodium phosphate [Na_2_HPO4], pH adjusted to 6.8). Muscle tissues were fixed in this solution for 48 to 72 h at RT, then rinsed once in PBS and transferred to 25 mL of 70% ethanol (vol/vol in distilled water) for transport to Adelaide Histology Services (University of Adelaide AHMS, Adelaide, Australia). Fixed tissues were embedded in paraffin blocks, sectioned, and stained with hematoxylin and eosin for histological analysis. Slides were imaged and scored by Adelaide Microscopy Services (University of Adelaide AHMS, Adelaide, Australia).

### Live Spn challenge.

Spn strains D39 (serotype 2), ST232 (serotype 3), P9 (serotype 6A), and 22-4597 (serotype 22F, SSI 22F/2, lot 06-06-52, NSW Health Pathology Westmead,) were grown in serum broth to OD_600_ = 0.2, then frozen at −80°C until the day of challenge. Mice were immunized as described earlier, then were IN challenged with live Spn 2 weeks after the final immunization. All mice were first anaesthetized by IP injection of ketamine anesthetic (10% ketamine + 1% xylazil vol/vol) suspended in injection-grade H_2_O, dosed at 10 μL per gram of animal body weight. Once anaesthetized, Spn inoculum containing 2 × 10^7^ CFU (ST232) or 10^8^ CFU (D39, P9, 22-4597) in 25 μL of PBS was administered through the nostrils. Mice were individually monitored to ensure regular breathing and recovery from anesthetic. For analysis of survival, mice were monitored multiple times daily for development of clinical symptoms for 2 to 3 weeks postchallenge and were humanely euthanized once moribund.

### Passive protection assay.

Pooled serum from rabbits immunized with Gamma-PN, Gamma-PN+Alhydrogel or prebleed serum was heat-inactivated at 56°C for 30 min. Pooled serum was mixed at a 1/10 dilution with Spn serotype 6A strain P9 (1 × 10^5^ CFU/mL) and incubated for 30 min at 37°C to allow antibodies to bind bacteria. Then, 100 μL of serum-treated Spn was injected IP into 4 to 6-week-old female Swiss mice (1 × 10^4^ CFU/mouse). All mice were monitored for 7 days postchallenge for clinical symptoms and overall survival.

### Protein array analysis.

Immune sera from Gamma-PN and Gamma-PN+Alhydrogel immunized mice or rabbits were pooled within each group in equal volumes. Sera from PBS mock-immunized mice were pooled as the mouse negative control, and prebleed sera from immunized rabbits were pooled as the rabbit negative control. Pooled sera were used in technical triplicate to probe a protein array containing 289 individual Spn proteins (61). Proteins were selected based on presence and conservation level in a panel of >600 Spn strains, and identification by Croucher et al. (61) as being recognized at significant levels by IgG present in human sera from healthy adults. Protein array analysis was performed as previously described by Schreeg et al. (62) and Nakajima et al. (63). Briefly, the array was constructed using genes from Spn strain TIGR4 where proteins were expressed and tested for expression by Western blotting using antibodies against N-terminal polyhistidine (His) tags and printed onto nitrocellulose-coated glass AVID slides (Grace Bio-Labs) using an Omni Grid 100 microarray printer (Genomic Solutions). Serum was diluted 1/100 in protein array blocking buffer (Maine Manufacturing, Sanford, ME) and supplemented with E. coli lysate. Images were acquired and analyzed using an ArrayCAM imaging system from Grace Bio-Labs. All results presented are expressed as median fluorescence intensity (MFI, ±SD) (62, 63). Antigens were assigned a positive result if all three replicates showed values greater than 2 SDs higher than values obtained for negative-control samples.

### Opsonophagocytic killing assay (OPKA).

Individual rabbit sera collected after the final immunization were tested for opsonophagocytic activity using the established OPKA protocol from the Bacterial Respiratory Pathogen Reference Laboratory (UAB, OPKA version E.02; available at: https://www.vaccine.uab.edu). This protocol was implemented as described, with the exception that Spn strains were tested individually rather than multiplexed. For flow cytometry confirmation of HL-60 cell differentiation, 5 mL of differentiated and nondifferentiated cells were washed in PBS and added to a 96-well U-bottom tray at 2 × 10^5^ cells/well. Cells were pelleted at 350 × *g* for 5 min, resuspended in 50 μL of near-infrared 780 fixable dye (BD, 1/1,000 dilution in PBS), incubated for 15 min at RT, then washed with Annexin V binding buffer (BD Pharmingen, catalog no. 556454) and resuspended in 50 μL anti-Annexin V antibody (BD Pharmingen, component no.51-65874X; diluted 1/20 in Annexin V binding buffer). Plates were incubated for 15 min at RT. Cells were washed in fluorescence-activated cell sorter (FACS) buffer and incubated in sterile human sera (diluted 1/50 in FACS buffer) to block human Fc receptors. Cells were washed with FACS buffer, then incubated on ice for 30 min with either: antihuman CD35, antihuman CD71 (both 1/60 final dilution in FACS buffer), or antihuman CD11b (final dilution 1/30), detailed in Table S1. Cells were washed in FACS buffer, then in PBS + 0.04% sodium azide before resuspension in 1% PFA. Data acquisition for all samples was performed on a BD LSR Fortessa X-20 flow cytometer. HL-60 differentiation was confirmed by flow cytometry, as described in Fig. S3-S5.

10.1128/mbio.02367-22.6 TABLE S1 Fluorophore-conjugated antibodies used in flow cytometry staining of HL-60 cells for OPKA. Download Table S1, DOCX file, 0.01 MB. Copyright © 2022 David et al. 2022 David et al. https://creativecommons.org/licenses/by/4.0/ This content is distributed under the terms of the Creative Commons Attribution 4.0 International license.

10.1128/mbio.02367-22.3 FIG S3 Gating Strategy to confirm HL-60 differentiation. HL-60 cells were differentiated for 5 days at 37°C in RPMI media supplemented with 10% FBS, 1% l-Glutamine and 0.8% N,N-Dimethylformamide (DMF). Additional cells were incubated in this media without DMF for 5 days to serve as nondifferentiated controls for flow cytometry analysis. After staining, cells were then washed and resuspended in 1% PFA for data acquisition, performed using a BD LSR Fortessa X-20 flow cytometer. Macrophage HL-60 cells were initially gated based on size, and doublets excluded by three successive single-cell gates as shown above. Single cells were gated based on 780 Fixable viability stain (APC-Cy7) and AnnexinV staining (APC) to determine viability, as further described in Fig. S4. Cells determined to be viable by 780 Fixable viability stain were subsequently gated on surface expression of CD11b, CD35, or CD71 (PE, each included in separate staining reactions), to determine success of HL-60 cell differentiation. This is described further in Fig. S5. Download FIG S3, TIF file, 2.3 MB. Copyright © 2022 David et al. 2022 David et al. https://creativecommons.org/licenses/by/4.0/ This content is distributed under the terms of the Creative Commons Attribution 4.0 International license.

10.1128/mbio.02367-22.4 FIG S4 Comparison of differentiated and nondifferentiated HL-60 control cells by viability analysis. Differentiated and nondifferentiated HL-60 cells were stained and acquired by flow cytometry as described in Fig. S3. Single cells were gated on 780 Fixable viability stain (APC-Cy7) and AnnexinV (APC) for viability analysis. The top two panels show gating on 780 fixable viability dye for differentiated and nondifferentiated cells. The bottom two panels show 780 viability gating against AnnexinV gating for differentiated and nondifferentiated cells. Unstained cells were used to set all gates, also ensuring <4% of stained cells appeared in Q1 (780 stain^+^, AnnexinV-). To be considered successfully differentiated and thus suitable for OPKA, ≥65% of differentiated HL-60 cells must be viable by 780 fixable viability staining and ≤25% of differentiated HL-60 cells can be in early apoptosis (780 stain-, AnnexinV^+^). Download FIG S4, TIF file, 2.3 MB. Copyright © 2022 David et al. 2022 David et al. https://creativecommons.org/licenses/by/4.0/ This content is distributed under the terms of the Creative Commons Attribution 4.0 International license.

10.1128/mbio.02367-22.5 FIG S5 Comparison of successfully differentiated and nondifferentiated HL-60 control cells by surface marker analysis. Differentiated and nondifferentiated HL-60 cells were stained and acquired by flow cytometry as described in Fig. S3. Single cells were gated on 780 fixable viability stain (APC-Cy7), and live cells only were subsequently gated on expression of surface markers CD11b, CD35, and CD71 (all on PE, each included in separate staining reactions). Unstained control cells were used to set the PE^+^ gate, ensuring this single gate was suitable for both nondifferentiated (top row) and differentiated (bottom row) unstained control cells (left-most panel in each row). To be considered successfully differentiated and thus suitable for OPKA, differentiated HL-60 cells must have up-regulated CD11b relative to nondifferentiated control HL-60 cells, ≥55% of differentiated HL-60 cells should be CD35^+^, and ≤20% of differentiated HL-60 cells should be CD71^+^. Download FIG S5, TIF file, 2.8 MB. Copyright © 2022 David et al. 2022 David et al. https://creativecommons.org/licenses/by/4.0/ This content is distributed under the terms of the Creative Commons Attribution 4.0 International license.

Spn strains for use in OPKA were grown as described for the IgG binding assay. Strains were diluted to 1 × 10^5^ CFU/mL in opsonophagocytic buffer B (OBB) and kept on ice until use. Rabbit sera were heat-inactivated, serially diluted with OBB, and mixed with bacteria. Trays were incubated for 30 min at RT in room air on a miniorbital shaker (350 rpm). Baby rabbit complement (complement, Pel-Freez) and differentiated HL-60 cells (E:T ratio 400:1) were added. Plates were incubated for 45 min at 37°C with 5% CO_2_ on an orbital shaker (350 rpm). Opsonophagocytosis was stopped by placing the plates on ice for 20 min. Samples were plated on blood agar plates, with incubation overnight at 37°C, 5% CO_2_ (30°C for serotype 3 to avoid overgrowth) prior to CFU enumeration. OPKA titers were calculated using the Opsotitre3 program (provided by Robert Burton, UAB). All sera were tested individually in the OPKA, except for the prebleed control samples which were pooled from all rabbits within a given immunization experiment. Rabbit sera from each immunization experiment were also tested in independent OPKA runs. This approach was required to save unnecessary use of animals (i.e., pilot immunization and OPKA testing was conducted for efficacy confirmation *prior* to commencement of second immunization experiment).

OPKA third-party validation was performed in the New Vaccines Laboratory, Murdoch Children’s Research Institute, Melbourne, Australia as previously published (64). The method used was as specified above with the following alterations: pooled serum from rabbits immunized with Gamma-PN or PCV13 were used, with 007sp reference serum used as a positive control. S. pneumoniae strains TREP6A and TREP22F (BEI Resources) were used in this study. Samples were plated on THY agar and allowed to absorb into plate. Once plates were dry, they were covered with an overlay containing THY agar + triphenyl tetrazolium chloride (TTC). After overnight incubation colonies were enumerated using a ProtoCOL3 colony counter.

### Statistical analyses.

Quantitative results were expressed as mean ± SD. One-way ANOVA was used for comparison of data from 3 or more groups involving a single independent variable, Two-way ANOVA was used when data were grouped according to two independent variables. Tukey’s multiple-comparison test was used for *post hoc* analysis. For nonparametric data (determined using the D'Agostino & Pearson test and the Shapiro-Wilk test), the Mann-Whitney U-test was used. Survival percentages were analyzed by Fisher Exact test or Mantel-Cox test. All analyses were performed using GraphPad Prism, version 9.0.1 (GraphPad Software, La Jolla, USA). *P*-values <0.05 (95% confidence) were considered statistically significant.

### Data availability.

Biological data relating to this study have been deposited in the community-approved and cross-disciplinary public repository Zenodo, under the DOI 10.5281/zenodo.7074253. All deposited data is open access, in accordance with findable accessible, interoperable, and reusable (FAIR) data principles.
