# Supplementary material for: A Nonadjuvanted Whole-Inactivated Pneumococcal Vaccine Induces Multiserotype Opsonophagocytic Responses Mediated by Noncapsule-Specific Antibodies
Source: mBio. 2022 Sep 20;13(5):e02367-22. doi: 10.1128/mbio.02367-22 (PMC9600166; doi:10.1128/mbio.02367-22)
Supplement: TABLE S3 [file mbio.02367-22-s0003.docx]

| **Subclass Ratio** | **Gamma-PN** | **Gamma-PN+Al** |
| --- | --- | --- |
| IgG1 : IgG2a | 2.66 (± 0.37) | 202.36* (± 164.33) |
| IgG1 : IgG2b | 2.92 (± 0.86) | 3.98 (± 2.82) |
| IgG1 : IgG3 | 6.29 (± 2.07) | 86.59 (± 57.45) |
