# Supplementary figures and images for: A Nonadjuvanted Whole-Inactivated Pneumococcal Vaccine Induces Multiserotype Opsonophagocytic Responses Mediated by Noncapsule-Specific Antibodies
Source: mBio. 2022 Sep 20;13(5):e02367-22. doi: 10.1128/mbio.02367-22 (PMC9600166; doi:10.1128/mbio.02367-22)

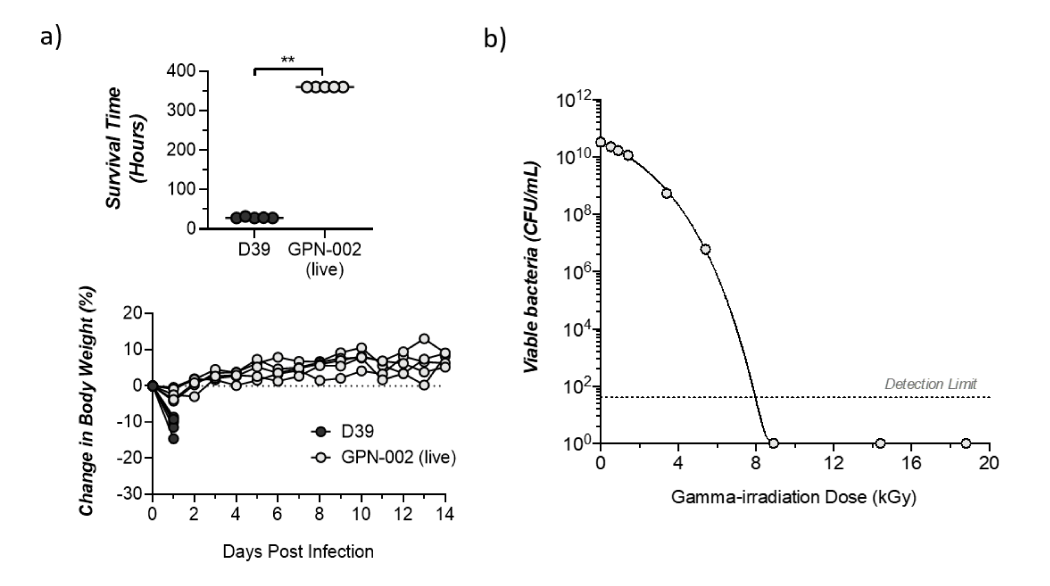

Supplement: FIG S1 [file mbio.02367-22-s0004.tif]

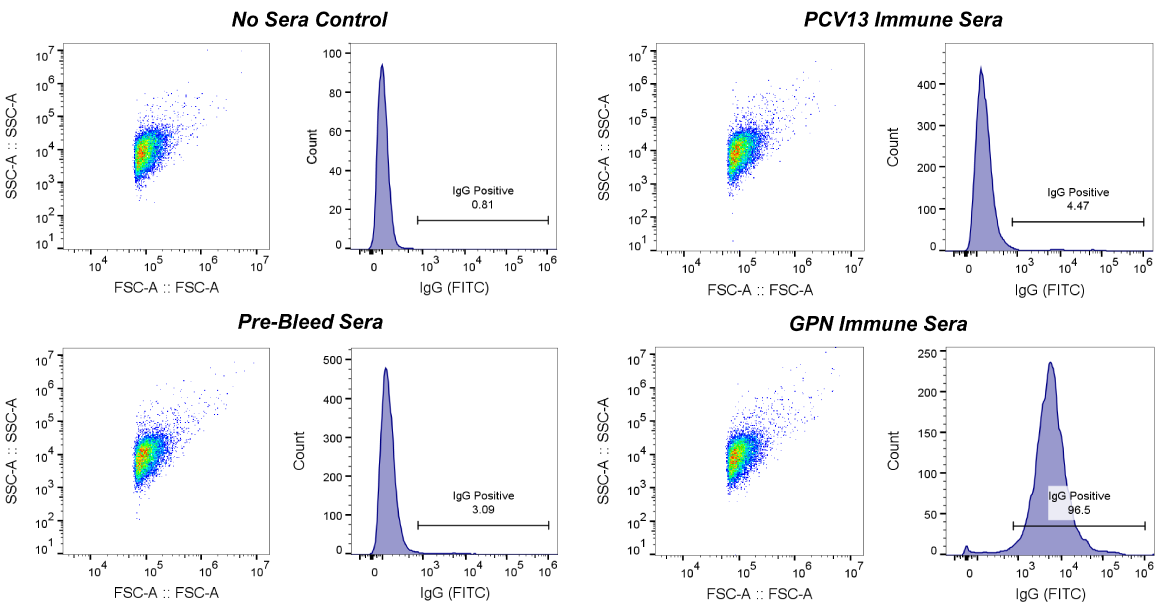

Supplement: FIG S2 [file mbio.02367-22-s0005.tif]

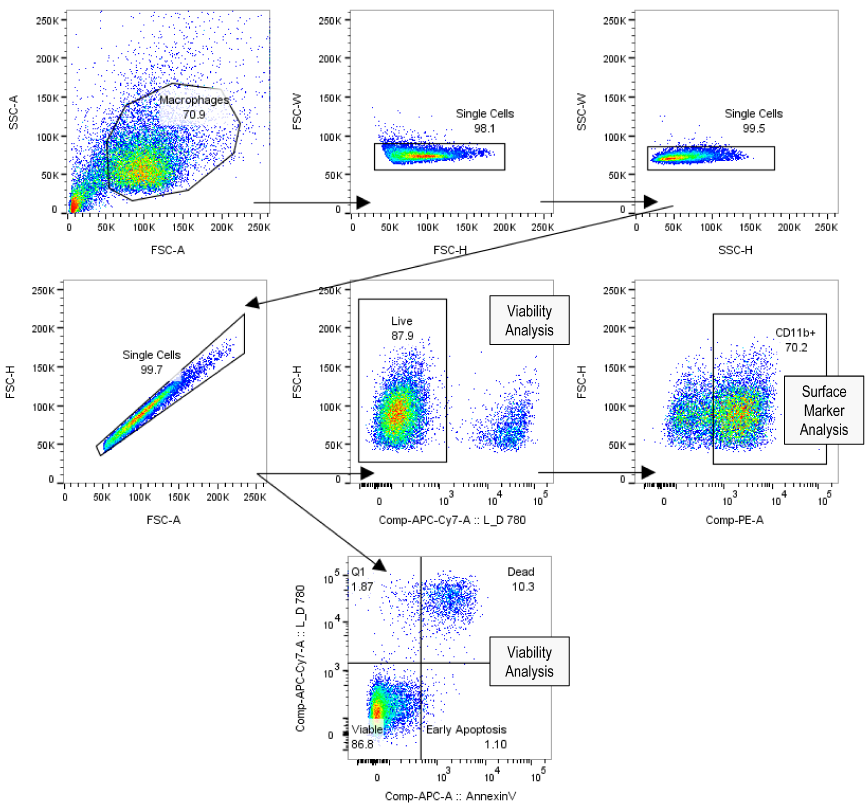

Supplement: FIG S3 [file mbio.02367-22-s0006.tif]

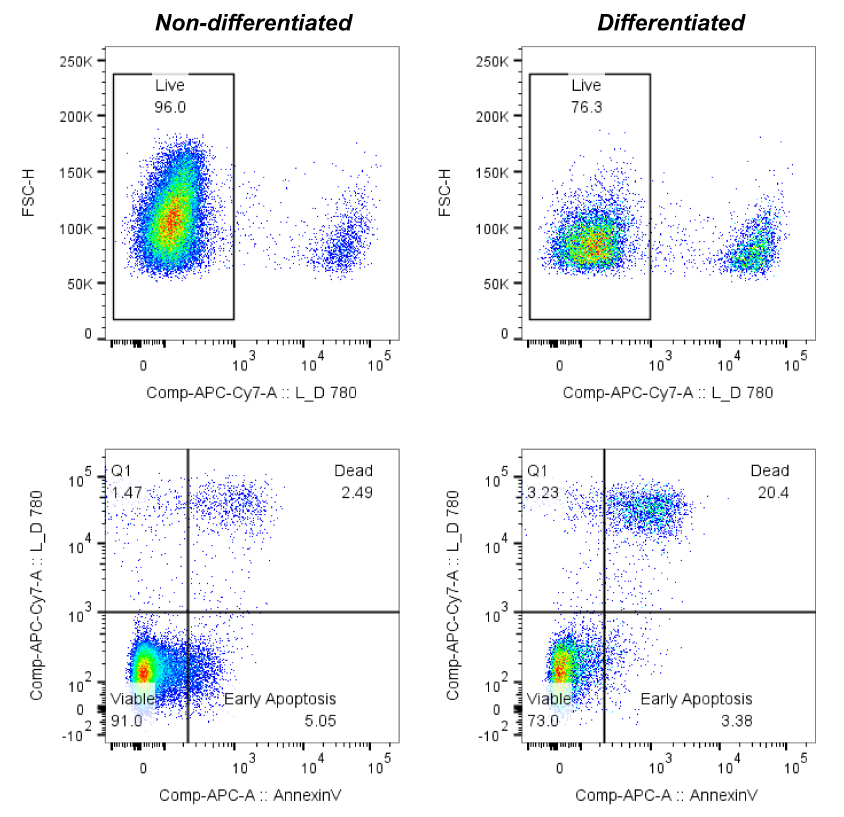

Supplement: FIG S4 [file mbio.02367-22-s0007.tif]

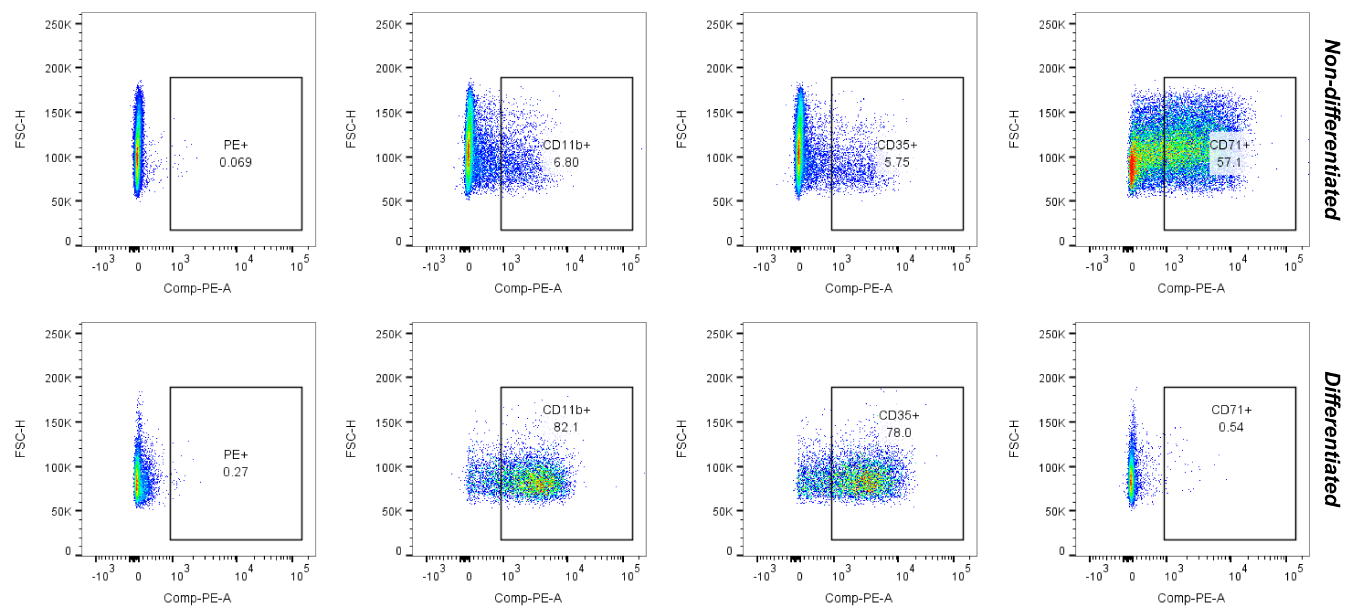

Supplement: FIG S5 [file mbio.02367-22-s0008.tif]
