# Supplementary material for: A Nonadjuvanted Whole-Inactivated Pneumococcal Vaccine Induces Multiserotype Opsonophagocytic Responses Mediated by Noncapsule-Specific Antibodies
Source: mBio. 2022 Sep 20;13(5):e02367-22. doi: 10.1128/mbio.02367-22 (PMC9600166; doi:10.1128/mbio.02367-22)
Supplement: TABLE S2 [file mbio.02367-22-s0002.docx]

| **Media Component** | **Amount** | **Units** | **Final Vol.** | **Supplier & cat. number** |
| --- | --- | --- | --- | --- |
| Soytone | 40 | g | 1L | BD 243620 |
| Yeast extract | 20 | g | 1L | Merck 1083469029 |
| Glucose | 20 | g | 1L | Thermo Fisher LP0021R |
| K_2_HPO_4_ | 5 | g | 1L | Merck 1048711000 |
| NaHCO_3_ | 1 | g | 1L | Sigma P2222 |
| L-glutamine | 0.624 | g | 1L | Sigma G8540 |
| L-asparagine | 0.1 | g | 1L | Sigma A7094 |
| MgSO_4_.7H_2_O | 0.5 | g | 1L | Sigma 63138 |
| 10 g/L FeSO_4_.7H_2_O | 0.5 | ml | 1L | Sigma M8179-100G |
| 10 g/L ZnSO_4_.7H_2_O | 0.08 | ml | 1L | Sigma 12354 |
| 10 g/L MnSO_4_.H_2_O | 0.036 | ml | 1L | Merck 1088811000 |
| 10 g/L choline chloride | 1 | ml | 1L | Sigma PHG0021 |
| TGA (98% solution) | 0.102 | ml | 1L | Sigma T3758 |
