# Supplementary material for: A Nonadjuvanted Whole-Inactivated Pneumococcal Vaccine Induces Multiserotype Opsonophagocytic Responses Mediated by Noncapsule-Specific Antibodies
Source: mBio. 2022 Sep 20;13(5):e02367-22. doi: 10.1128/mbio.02367-22 (PMC9600166; doi:10.1128/mbio.02367-22)
Supplement: TABLE S1 [file mbio.02367-22-s0001.docx]

| **Target (species)** | **Fluorophore/Conjugate** | **Supplier & cat. number** |
| --- | --- | --- |
| *HL-60 Differentiation* | | |
| CD35 (human) | PE | BD 559872, clone E11 |
| CD71 (human) | PE | BD 555537, clone M-A712 |
| CD11b/Mac-1 (human) | PE | BD 555388, clone ICRF44 |
| Annexin V (mammalian) | APC or FITC | Biolegend 640920; BD 51-65874X |
